# Cholinergic modulation of cognition: Insights from human pharmacological functional neuroimaging

**DOI:** 10.1016/j.pneurobio.2011.06.002

**Published:** 2011-09-01

**Authors:** Paul Bentley, Jon Driver, Raymond J. Dolan

**Affiliations:** a Wellcome Centre for Neuroimaging at UCL, University College London, 12 Queen Square, London WC1N 3BG, UK; b Department of Clinical Neuroscience, Charing Cross Hospital, Imperial College London, Fulham Palace Rd., London W6 8RF, UK; c UCL Institute of Cognitive Neuroscience, University College London, 17 Queen Square, London WC1N 3AR, UK

**Keywords:** ACh, acetylcholine, BOLD, blood-oxygen level dependent, CS, conditioned stimuli, EEG, electroencephalography, fMRI, functional magnetic resonance imaging, LTP, long-term potentiation, MEG, magnetoencephalography, PET, positron emission tomography, rCBF, regional cerebral blood flow, Cholinergic, Pharmacological, Functional imaging, fMRI, PET, Attention, Memory, Sensory

## Abstract

Evidence from lesion and cortical-slice studies implicate the neocortical cholinergic system in the modulation of sensory, attentional and memory processing. In this review we consider findings from sixty-three healthy human cholinergic functional neuroimaging studies that probe interactions of cholinergic drugs with brain activation profiles, and relate these to contemporary neurobiological models. Consistent patterns that emerge are: (1) the direction of cholinergic modulation of sensory cortex activations depends upon top-down influences; (2) cholinergic hyperstimulation reduces top-down selective modulation of sensory cortices; (3) cholinergic hyperstimulation interacts with task-specific frontoparietal activations according to one of several patterns, including: suppression of parietal-mediated reorienting; decreasing ‘effort’-associated activations in prefrontal regions; and deactivation of a ‘resting-state network’ in medial cortex, with reciprocal recruitment of dorsolateral frontoparietal regions during performance-challenging conditions; (4) encoding-related activations in both neocortical and hippocampal regions are disrupted by cholinergic blockade, or enhanced with cholinergic stimulation, while the opposite profile is observed during retrieval; (5) many examples exist of an ‘inverted-U shaped’ pattern of cholinergic influences by which the direction of functional neural activation (and performance) depends upon both task (e.g. relative difficulty) and subject (e.g. age) factors. Overall, human cholinergic functional neuroimaging studies both corroborate and extend physiological accounts of cholinergic function arising from other experimental contexts, while providing mechanistic insights into cholinergic-acting drugs and their potential clinical applications.

## Highlights

• We review all 63 healthy human cholinergic functional neuroimaging studies to date. • General findings from fMRI and PET studies are synthesised with biological models. • Cholinergic neuromodulations are divided into sensory, attentional or memory. • Methodological issues of pharmacological functional imaging are discussed.

## 1 Introduction

Disruption to cholinergic neurotransmission – whether by targeted lesions, toxins, drugs, aging or disease –induces impairments in a range of functions, including perception (Erskine et al., 2004), attention (Robbins et al., 1989), memory and learning (Kopelman, 1986), emotion (Kamboj and Curran, 2006), and sleep (Kim and Jeong, 1999). This broad cognitive-behavioural profile reflects in part the pan-cortical and subcortical reach of cholinergic neurons (Mesulam and Geula, 1991). These facts, together with evidence for *en masse* activation of corticopetal cholinergic fibres (Phillis and Chong, 1965); a non-synaptic, ‘volume transmission’ mode of acetylcholine release (Descarries et al., 1997); generalised postsynaptic excitation (Krnjević et al., 1971); plus cholinergic correlations with EEG desynchronisation (Buzsàki and Gage, 1989) and gamma rhythms (Börgers et al., 2005), have led to one conceptualisation of acetylcholine (ACh) as part of an ascending arousal system (Lewandowski et al., 1993; Berntson et al., 2003) that potentiates consciousness (Frohlich and Franco, 2010).

In contrast to the role of ACh as a diffuse neural activator, a growing body of evidence demonstrates that cholinergic transmission can also act on more precise spatial and temporal scales (Sarter et al., 2009). Cholinergic influences in non-human primates are often synaptically localised (Smiley et al., 1997), regionally segregated (Lidow et al., 1989), and modulated over narrow spatial ranges by virtue of fractional basal forebrain activation (Golmayo et al., 2003); local glutamatergic control of cholinergic terminals (Parikh et al., 2008); or cortical cholinergic interneurons (von Engelhardt et al., 2007). Phasic, as opposed to tonic, cholinergic signalling may engender predominantly inhibitory, rather than excitatory, postsynaptic effects (Gulledge et al., 2007), thereby altering spatial patterns of cortical activity (Xiang et al., 1998; Roberts et al., 2005). These emerging properties of the cholinergic system suggest that ACh sculpts information-flow in ways that favour specific rather than diffuse processing. For example, by favouring stronger, at the expense of weaker, inputs (Krnjević et al., 1971; McCormick and Prince, 1986), or by selective strengthening of input-driven synapses (Huerta and Lisman, 1993), the cholinergic system may bias particular modes of attentional or memory processing, respectively (Hasselmo and McGaughy, 2004).

To reconcile these two roles – as a general modulator of cortical activity, and as a mediator of highly specific regional processing effects – several models have emerged that embrace both perspectives (e.g. Hasselmo and Sarter, 2011; Sarter et al., 2005; Yu and Dayan, 2005). Such models typically characterise the cholinergic system as an orchestrator of neural activity across widespread cortical fields, with separable cholinergic influences on sensory processing, attention and memory, all acting in synergy. Furthermore, characteristic patterns of cholinergic neural modulation on columnar circuitry (Hasselmo and Cekic, 1996) and cortical oscillations (Rodriguez et al., 2004), appear to be replicated in diverse parts of the cortex, with differing functional effects depending upon each region's connectivity.

Since acetylcholine acts over broad populations of neurons, causing fundamental shifts in processing modes or cortical patterning (e.g. Kilgard and Merzenich, 1998; Kimura et al., 1999), we might expect that functional neuroimaging – by virtue of its ability to map cortical activations associated with sensory, attentional and memory processes – should be sensitive to cholinergic manipulations. Over the last eighteen years, in the order of one hundred functional neuroimaging studies have been published, in healthy humans and patients, that describe brain activation patterns associated with administration of drugs acting on cholinergic pathways, in different cognitive contexts.

The purpose of the current review is to evaluate how human functional neuroimaging contributes to an understanding of cholinergic interactions with cognitive function. The review focuses on fMRI or PET studies in healthy subjects who received either cholinergic antagonists or stimulants, and who were scanned during active task states (as opposed to solely resting). A comprehensive list of all sixty-three human cholinergic functional imaging studies is tabulated, with results divided according to whether modulations are primarily within sensory, frontoparietal or medial temporal cortices, and according to the cognitive function tested. We subsequently attempt a synthesis of general patterns of cholinergic neuromodulation, and suggest neural bases for these given contemporary integrative models. In order to facilitate interpretation, we firstly provide an overview of existing cholinergic neurophysiology (i.e. derived primarily from non-human or human behavioural pharmacology studies), before considering pharmacological functional neuroimaging methodological issues of relevance.

## 2 Cholinergic modulation of cognitive processing – non-human and psychopharmacological studies

### 2.1 Sensory

Sensory cortices are richly innervated by cholinergic fibres from nucleus basalis (Zilles and Wree, 1990), and show some of the highest regional densities of cholinergic receptors across the cortex (Zilles et al., 2002). The importance of this input is demonstrated by a profound impairment in stimulus sensitivity that follows cholinergic denervation (Sato et al., 1987a). Moreover, cholinergic release within sensory areas is triggered by stimulus presentation in a modality-specific manner (Laplante et al., 2005; Fournier et al., 2004), and is enhanced by directing attention to particular stimulus properties in awake animals (Sarter et al., 2005). More complex sensory functions, such as feature binding and selective attention, also involve neocortical cholinergic afferents, but appear more dependent upon cholinergic influences in frontoparietal regions (Botly and De Rosa, 2009).

Sensory cortices are highly responsive to acetylcholine (ACh), for example with ∼90% of visual cortical neurons showing responses to ACh, of which approximately two-thirds show facilitation (Sato et al., 1987b; Zinke et al., 2006). However, response patterns appear segregated, with cholinergic facilitation of cortical activity occurring in cells that: (1) are confined to layer IV, i.e. receive input from lower stages of sensory processing, including sensory afferents via the thalamus (e.g. in somatosensory cortex: Gil et al., 1997; or visual cortex: Kimura et al., 1999; or auditory cortex: Hsieh et al., 2000); and (2) are already strongly driven by input activity (Krnjević and Phillis, 1963). Sensory neurons that reside in other layers – receiving intracortical inputs – plus layer IV cells that are not co-stimulated by sensory inputs, are suppressed rather than facilitated by ACh (Sato et al., 1987b; Kimura et al., 1999).

As a consequence of such selective facilitation/suppression, ACh can increase signal-to-noise ratio, as well as reduce top-down and lateral influences on sensory responses. These effects in turn are manifest as enhanced stimulus detectability (Sato et al., 1987b), while reducing influences of spatial context (Roberts et al., 2005) or expectation (Yu and Dayan, 2005). Furthermore, by reducing intracortical lateral inhibition, and favouring anatomically non-dominant over dominant sensory inputs (Kuo et al., 2009), ACh can broaden sensory tuning curves and thereby reduce stimulus selectivity (Sato et al., 1987b; Zinke et al., 2006). Although this might be expected to impair certain sensory functions, e.g. stimulus discrimination, other studies have observed that raised ACh levels can sharpen sensory tuning curves (Sillito and Kemp, 1983; Murphy and Sillito, 1991), the discrepancy possibly arising from differences in the type of neurons sampled (Zinke et al., 2006), ACh stimulation pattern or animal species.

Acetylcholine also prolongs stimulus-evoked responses by reducing adaptation (McCormick and Prince, 1986; Zinke et al., 2006); potentiates subsequent responses for up to 30 min or so (Golmayo et al., 2003), and promotes gamma-range synchronisation of sensory units (Rodriguez et al., 2004). In turn, these effects can increase the likelihood that a stimulus will be detected and correctly discriminated (Sato et al., 1987b; Womelsdorf et al., 2006), and facilitate encoding into memory through mechanisms such as long-term potentiation (in sensory cortices: Bröcher et al., 1992; Dringenberg et al., 2007; and medial temporal cortices: Huerta and Lisman, 1993), and plastic reshaping of sensory cortex maps (Weinberger, 2007). Thus, many of the described cholinergic influences on sensory cortices are conducive to both attentional enhancement of sensory processing, as well as memory encoding (Golmayo et al., 2003; Sarter et al., 2005), thereby favouring processing of stimuli recently encountered (Greuel et al., 1988; Gu, 2003; Hasselmo and Sarter, 2011), and/or those accorded emotional significance (Weinberger, 2007).

### 2.2 Attention

Neocortical cholinergic lesions impair the ability to detect, identify, or localise brief stimuli, especially in the presence of attention-demanding challenges such as distractors, while not affecting overall motivational state, response rate, rule memory, or directional bias (Robbins et al., 1989; Moore et al., 1995; Muir et al., 1994). The fact that such lesions result in performance impairments that are proportionate to the degree to which sensory/attentional processing is taxed (Himmelheber et al., 2001), suggests that the cortical cholinergic system plays a role in shaping interactions of attention with sensory processing, rather than influencing either in isolation (McGaughy et al., 2002). One influential model relates neocortical cholinergic release with the degree of mismatch between motivation-driven goals and actual performance, i.e. ‘attentional effort’ (Sarter et al., 2006). By this means, cortical ACh levels increase following challenges that degrade reward-driven performance, which itself is instrumental in reversing the initiating behavioural impairment (Himmelheber et al., 2000; Kozak et al., 2006). This may account for correlations between ACh release and either sensory demands or motor response (Richardson and DeLong, 1990; Passetti et al., 2000).

The functional anatomy (and effective connectivity) by which the cortical cholinergic system supports attention involves interactions between prefrontal, parietal and sensory regions (Golmayo et al., 2003; Nelson et al., 2005). Performance monitoring information from prefrontal regions, combined with arousal and motivational information from reticular and limbic regions, provides input to basal forebrain, and determines cortical acetylcholine release (Sarter et al., 2006; Gozzi et al., 2010). The amygdala may also directly activate basal forebrain, in conveying contingency-violation or fear-conditioned signals (Holland and Gallagher, 2006). In turn, cholinergic inputs to prefrontal and parietal regions modulate processes such as distractor suppression (Gill et al., 2000), attentional shifting (Davidson and Marrocco, 2000) and disengagement (Bushnell et al., 1998) between spatial locations or features (Bucci et al., 1998). Following repeated training with an attention-taxing task, cellular mediators of cholinergic neurotransmission are upregulated in prefrontal regions, and correlate with enhanced signal detection (Apparsundaram et al., 2005). Cholinergic inputs to prefrontal cortex may also serve to inhibit impulsive responses via subcortical structures (Bushnell et al., 1998; McGaughy et al., 2002).

Cholinergic influences on *bottom-up* sensory processing – including selectively potentiating stimulus-evoked inputs and suppressing adaptation (Section 2.1) – complement effects of ACh on *top-down* attentional shifting and focusing (Sarter et al., 2001). This is supported by evidence that ACh is released in a pan-cortical fashion (Phillis and Chong, 1965), and that selective attention is dependent upon cholinergic stimulation of both frontoparietal (Gill et al., 2000; Broussard et al., 2009) and sensory cortices (Herrero et al., 2008).

A computational account of cortical acetylcholine release relates it to processing ‘uncertainty’ (Yu and Dayan, 2005) regarding stimulus–stimulus or stimulus–response contingencies (Bucci et al., 1998; Dalley et al., 2001). On this view, high acetylcholine levels favour bottom-up over top-down processes, so as to reduce cortical inference in times of uncertainty (see also Hasselmo, 1995). Importantly, this model accords with ACh efflux being related both to ‘attentional effort’ in the face of performance challenges (Arnold et al., 2002) and to novelty (Acquas et al., 1996; Wilson and Rolls, 1990). It also fits cortical slice data demonstrating that ACh promotes feedforward over feedback signalling (Hasselmo and McGaughy, 2004). The model successfully predicts that cholinergic levels are inversely correlated with cue validity in a Posner spatial-attention paradigm, and that as ACh levels increase, the degree to which a cue focuses attention – i.e. the cue validity effect – decreases (Phillips et al., 2000). Furthermore, prefrontal ACh innervation mediates cognitive flexibility during serial contingency reversals, but not initial acquisition of contingency (Cabrera et al., 2006), consistent with ACh communicating expected, rather than unexpected, uncertainty – the latter of which may be represented by norepinephrine instead (Yu and Dayan, 2005).

### 2.3 Memory

Memory impairment is strongly associated with cholinergic receptor antagonism, or cholinergic neuropathology, as for example seen early in Alzheimer's disease (Kopelman, 1986). The hippocampus contains one of the highest cerebral densities of cholinergic fibres (Mesulam et al., 1986), and receives dedicated cholinergic input from the septal basal forebrain, distinct from the nucleus basalis–neocortical projection. While this anatomical split of the cholinergic system into neocortical and hippocampal divisions may roughly underlie cholinergic mediation of attention and memory, respectively (Everitt and Robbins, 1997), accumulating evidence suggests that the neocortical cholinergic innervation is also important for normal memory (Hasselmo and McGaughy, 2004; Parent and Baxter, 2004).

A key behavioural finding is that acetylcholine is more critical for memory encoding than consolidation (Hasselmo, 1999), with cholinergic stimulation being counterproductive if occurring *after* encoding (Bunce et al., 2004; Gais and Born, 2004). A characteristic of cholinergic modulation on columnar circuitry (Gil et al., 1997) – described above in the context of sensory processing – may account for this directionality in time, arising not only in sensory, but also entorhinal and hippocampal cortices (Hasselmo and McGaughy, 2004). Specifically, ACh-induced favouring of feedforward connections encourages self associations between novel patterns of input, while suppression of feedback by ACh minimizes the risk of pro-active interference from previously established associations (Hasselmo, 1995; De Rosa et al., 2001; Atri et al., 2004). Conversely, consolidation of existing traces, and retrieval, are supported by a feedback-predominant state that occurs under low ambient ACh levels, e.g. slow-wave sleep. Differential impacts of ACh on encoding and retrieval components of memory may also be related to modulation of the hippocampal theta rhythm (Hasselmo et al., 2002).

As well as influencing feedforward-versus-feedback dynamics, ACh impacts upon several other cortical memory mechanisms. Two of these – long-term potentiation (LTP) and persistent-spiking – are expressed in higher sensory, entorhinal, perirhinal and hippocampal, as well as prefrontal, regions (Klink and Alonso, 1997; Anagnostaras et al., 2003; McGaughy et al., 2005; Hasselmo and Stern, 2006). Cholinergic potentiation of these may underlie observations that nucleus basalis activation acts as a driver for sensorimotor cortex remapping, e.g. as seen with changes in the representation of a conditioned auditory stimulus in tonotopic auditory cortex (Weinberger, 2007). This in turn may account for cholinergic influences on behaviours such as conditioning and motor learning (Conner et al., 2003). Cholinergic influences on LTP and persistent-spiking may also underlie the dependency of sensory discrimination learning and recognition memory on cholinergic inputs to extrastriate temporal and perirhinal cortices, respectively (Ridley et al., 2005; Tang et al., 1997). By contrast, repetition suppression (i.e. a reduced neural response for repeated relative to novel stimuli) has not been found to be dependent on cholinergic integrity in monkey inferior temporal cortex (Miller and Desimone, 1993), despite evidence that behavioural priming (i.e. an improvement in performance to repeated stimuli) is sensitive to cholinergic manipulation (Thiel et al., 2002c).

Numerous other examples exist of cholinergic memory effects that suggest additional mechanisms. These include ACh-induced prolongation of evoked responses and enhancement of signal-to-noise ratio in hippocampus (Everitt and Robbins, 1997); and cholinergic influences on prefrontal cortex for working memory (Chudasama et al., 2004); cingulate cortex for sensory-response contingency learning (Dunnett et al., 1989) and amygdala for consolidation (rather than acquisition) of contextual conditioning (Power, 2004).

## 3 Cholinergic-functional neuroimaging – methodological considerations

### 3.1 Cholinergic functional neuroimaging in humans

Functional neuroimaging has increasingly established itself as a valid and informative tool for studying activation patterns across the whole brain in different cognitive and/or pharmacological contexts, complementing invasive methodologies such as single-unit or lesion-based techniques. Such convergence has arisen even though most functional neuroimaging measures primarily reflect regional metabolic or vascular responses, as indirect indices of neural activity (Logothetis, 2002); and despite the restricted (millimetre) spatial resolution of existing functional neuroimaging approaches.

Numerous examples of functional neuroimaging paradigms exist that show robust, reproducible and interpretable regional activations, consistent with more invasive measures in animals. These include retinotopic (e.g. DeYoe et al., 1996) and category-specific (e.g. Kanwisher et al., 1997) mappings of visual cortex; attentional influences on sensory cortices (e.g. Martinez et al., 2001); attentional control signals in frontoparietal regions (e.g. Hopfinger et al., 2000); learning-related plasticity of sensory cortex (e.g. Morris et al., 1998); repetition suppression (e.g. Henson and Rugg, 2003; van Turennout et al., 2003); working memory-delay activity (e.g. Courtney et al., 1997); and subsequent-memory effects in medial temporal cortex (e.g. Wagner et al., 1999). From the standpoint of the current review, it is notable that many such functional imaging paradigms probe neural mechanisms that non-human studies have shown to be under cholinergic control. Consequently, it becomes meaningful to ask whether or not cholinergic manipulations alter activation patterns in directions consistent with existing accounts; and furthermore whether human neuroimaging can provide new data to help refine existing models of cholinergic function.

### 3.2 Possible confounding factors in pharmacological functional neuroimaging

With *pharmacological* functional neuroimaging, the gap between that which we wish to infer (i.e. neural activity) and that which is actually measured (typically regional cerebral blood flow (rCBF) or blood-oxygen level dependent (BOLD) magnetic resonance signal) becomes more complicated, due to potential pharmacological influences on the neurovascular relationship. Many types of drug influence both rCBF (Edvinsson et al., 1987) and coupling between blood flow and metabolism (Leithner et al., 2009) – both with the potential to confound fMRI and PET (Burke and Bührle, 2006). Furthermore, drug influences on baseline (i.e. resting-state) neural activity or blood flow may modulate the apparent strength and spatial spread of active functional responses (van Eijsden et al., 2009).

Of particular relevance here is that acetylcholine can act both to increase regional cerebral blood flow, and to uncouple neurovascular responses (Table 1); moreover, these effects may vary by brain region and age. For example, cholinergic antagonism with scopolamine suppresses the rCBF response to somatosensory stimulation, but does not affect glucose uptake (Ogawa et al., 1994). Conversely, cholinergic stimulation (e.g. with physostigmine) increases global CBF, apparently at the same time as not altering cerebral metabolism (Scremin et al., 1982; Hallstrom et al., 1990; Tsukada et al., 1997), or electrophysiological responses (Lacombe et al., 1989), or even while *decreasing* regional metabolism (Blin et al., 1997). It should be noted, however, that the doses of cholinergic drugs required to induce direct effects on vasomotor reactivity appear to be significantly greater than those typically used in functional neuroimaging studies, even when in the latter cases behavioural effects due to relatively low drug doses can be reliably observed (e.g. Thiel et al., 2001).

The extent to which cholinergic influences on cerebral blood flow confound cholinergic-functional imaging findings is unclear. Acetylcholine-induced increases of event-related rCBF responses would be expected to increase BOLD responses measured by fMRI (Davis et al., 1998), independent of any neural effects. Alternatively, if ACh increases baseline (i.e. resting) rCBF, then we might expect reduced BOLD responses, as can occur with acetazolamide administration or hypercapnia (Cohen et al., 2002; Brown et al., 2003). However, several studies report no changes in stimulus-induced BOLD or rCBF responses in early visual cortex secondary to nicotine (Jacobsen et al., 2002; Hahn et al., 2007); scopolamine (Thiel et al., 2001; Sperling et al., 2002), or physostigmine (Mentis et al., 2001; Furey et al., 2000a,b,c). Furthermore, perfusion-sensitive MRI shows no effect of nicotine on rCBF changes elicited by stimulus or movement in early sensory and motor areas, respectively (Hahn et al., 2009). These facts suggest a degree of immunity of typical functional neuroimaging measures, when combined with typical, relatively low drug doses, to cholinergic-vascular interactions – even in cortical regions where such interactions might be most prominent (Sato et al., 2004). For greater certainty, co-measurement of BOLD or rCBF with electrical activity is ideally needed. In this regard, it is reassuring to note that BOLD responses remain tightly coupled to local electrical activity following challenges with vasoactive drugs such as general anesthetics, serotonergic agonists and cocaine (Goense and Logothetis, 2008; Rauch et al., 2008; Gollub et al., 1998). Furthermore, the direction of cholinergic influences on task-related PET and fMRI activations in thalamus (typically enhanced by pro-cholinergic drugs: Cohen et al., 1994; Mentis et al., 2001) and visual cortex (typically suppressed by pro-cholinergic drugs: Bentley et al., 2004; Silver et al., 2008) appear congruent with cholinergic influences on electrophysiological responses in these same regions (McCormick, 1992; Lörincz et al., 2008; Kimura et al., 1999).

### 3.3 Strategies to circumvent possible confounds in pharmacological neuroimaging

Given concerns over pharmacological influences on the neurovascular coupling relationship, pharmacological functional imaging experiments are designed in ways that: (1) assess any changes in global and/or session parameters, independent of the cognitive paradigm, and (2) reduce the impact of any such potential confound. Table 2 outlines the range of methodological strategies employed in this regard, with examples given from the cholinergic functional imaging literature (see also Iannetti and Wise (2007) for methodological issues pertaining to pharmacological fMRI more generally).

Unlike resting-state activation studies (e.g. Honer et al., 1988; Geaney et al., 1990), task-related studies as focused on here probe for *interactions* between a drug challenge and two or more functional conditions. For example, if a given brain region shows approximately similar levels of activation across two or more behavioural conditions (relative to baseline), then finding that a drug modulates activity specifically during a subset of conditions, but not during others, strongly suggests an interaction with a neurocognitive process (e.g. Hahn et al., 2009). However, where activation strengths differ between conditions, before drug is given, then task × drug interactions may actually reflect effects on a neurovascular scaling relationship, or appear due to metabolic-vascular ceiling (or floor) effects. In these cases, it is useful to observe whether other neocortical regions showing a similar condition-specific pattern of activity, in the absence of drug, also demonstrate the same type of condition × drug interaction – suggesting perhaps a non-specific, vascular drug effect, or whether the interaction is region-specific (e.g. Thiel et al., 2001). However region-specific modulations provide only a partial guarantee for inferring neural modulations, not least because cholinergic drug effects on cerebral vasculature may vary between regions, with cholinergic stimulation increasing rCBF by progressively smaller amounts between prefrontal, parietal, hippocampal and subcortical regions (Adachi et al., 1990; Lacombe et al., 1989; Sato et al., 2004).

If it is assumed (or demonstrated) that drug-induced changes in our neuroimaging signal (e.g. rCBF or BOLD) reflect a specific neural effect, rather than a nuisance vascular effect, the question then arises as to what this modulated activity signifies. If a drug-induced modulation of regional activation is accompanied by a behavioural change, it is tempting to interpret the functional imaging change causally, in terms of enhanced or impaired functionality of the modulated region. However, one must also consider the possibility that the activation change might reflect compensation (e.g. for a remote drug-induced impairment in some other areas), or are a consequence of the observed behavioural change rather than vice versa. In certain situations, it is possible to make a reasonable interpretation of what drug-induced modulation of cerebral activity means even without concomitant behavioural changes, e.g. when increasing or decreasing top-down attentional-modulation of sensory activations (Bentley et al., 2004), or when biasing the relative sizes of sensory stimulus representations (Thiel et al., 2002a).

A further set of methodological considerations arises from the timeline for the pharmacological action of particular cholinergic drugs. Ideally, scanning should take place during a timewindow when pharmacokinetic and pharmacodynamic variables are relatively stable – the latter as established either chemically or behaviourally (Furey et al., 2000c). Animal microiontophoretic and human radioligand studies contribute in this regard by demonstrating, for example, the time-course of effects from cholinergic drugs on brain neurotransmitter levels (Tsukada et al., 2004) and receptor occupancy (Brody et al., 2006). In other cases, drug-induced functional activation changes may be assessed many days after the drug has been withdrawn, presumably due to drug influences on plasticity (Rosier et al., 1999). It also needs to be appreciated that the response to a set dose of a drug, or the dose at which an optimal behavioural effect is achieved, can vary widely between subjects for drugs such as cholinesterase inhibitors (Bartus, 2000). This may be related to age, baseline performance, and genetic variability (Kukolja et al., 2009; Bizzarro et al., 2005), which if known, can be included as covariates within the regression model. Finally, we note that the localisation of pharmacological functional neuroimaging effects will not necessarily reflect the *only* sites where the drug is acting. For example, enhancements of neocortical activation secondary to cholinergic stimulation may reflect modulatory effects within the thalamus, mediated via a tegmental-thalamic reticular system, rather than due to interactions with the nucleus basalis–neocortical system (McNamara et al., 1990). Furthermore, cholinergic drugs are likely to influence other neuromodulatory systems such as dopamine (Dewey et al., 1993), while muscarinic receptor blockers may paradoxically enhance cholinergic neurotransmission, e.g. within hippocampus (Mishima et al., 2000), due to autoregulatory effects (Hoss et al., 1990), or by enhancing nicotinic receptor transmission (Mentis et al., 2001).

## 4 Systematic review

We performed an exhaustive search for human cholinergic functional imaging studies using the PubMed database with combinations of the search terms [*cholinergic* OR *acetylcholine* OR *nicotine* OR *scopolamine* OR *cholinesterase* OR *smoking* OR *varenicline*] AND [*functional imaging* OR *fMRI* OR *PET*] up to May 2011. PubMed-suggested ‘Related Articles’, references and citations of relevant articles were also interrogated. Selected studies were those in which: (1) functional neuroimaging measures were obtained in healthy humans during a stimulus-driven and/or behavioural activation paradigm; and (2) the effects of a systemic cholinergic manipulation on brain activation patterns were examined. The majority of such studies actually scanned subjects over at least two behavioural conditions, sometimes including a resting state. Hence, the results of such studies often take the form of interactions between drug and task- (or stimulus-) determined conditions in determining regional brain activations.

Cholinergic functional neuroimaging studies in patient groups (mostly in Alzheimer's disease or mild cognitive impairment) are not listed here because of differences in the general methodology of such studies. The vast majority of such clinical studies observe changes in neural activation over a *long course* of treatment (typically many months), rather than using placebo-controlled, single drug challenges, unlike most healthy studies. Moreover, many measure resting-state metabolic profiles alone, rather than task and/or stimulation-related activations. Clinically focused cholinergic functional neuroimaging reviews can be found elsewhere (see Dickerson, 2006; Nordberg, 2004).

A summary of all relevant human cholinergic functional imaging studies is presented in Tables 3–5. To assist exposition, and in line with the various functional conceptualisations of acetylcholine summarised in Section 2 (viz. sensory, attention and memory functions), studies are categorised according to whether the critical effects primarily arose in sensory, frontoparietal or medial temporal cortical regions. Activations in other brain regions (e.g. lateral temporal cortex and subcortical structures) are listed alongside frontoparietal effects for convenience. Furthermore, within each anatomical division, effects are secondarily classified according to the broad cognitive construct putatively tested (e.g. passive viewing, attention-demanding or memory task). Then for each study and anatomical region we tabulate: a more accurate description of the behavioural paradigm; the drug administered; the imaging modality; plus the critical functional neuroimaging and behavioural results. Studies are duplicated across tables where, for example, both sensory and frontoparietal regions were studied.

The general format of all studies is that of a randomised-controlled trial in which subjects receive a single challenge or short course of drug or placebo. Most studies adopt a within-subject, cross-over design. Cholinergic drugs used were scopolamine (muscarinic receptor antagonist); nicotine (nicotinic receptor agonist); physostigmine or donepezil (cholinesterase inhibitors; the former of which is given intravenously and has a well-documented pharmacokinetic time-course); mecamylamine (nicotinic receptor antagonist), or varenicline (a nicotinic α4β2 receptor partial agonist and α7 receptor full agonist, typically used in the context of ameliorating smoking withdrawal symptoms; and in functional imaging paradigms often administered for 2–3 weeks before scanning). Studies in which smoking a cigarette are used as a nicotine source are also included, although such studies typically do not control for the behavioural or chemical properties of smoking, and so must be interpreted accordingly.

## 5 Functional neuroimaging: sensory modulations

### 5.1 Sensory cortex modulations depend upon task demands

#### 5.1.1 Functional neuroimaging results

Forty-one functional imaging studies in healthy adults have probed interactions of cholinergic modulation with stimulus-evoked sensory cortex activity (Table 3). Results are categorised according to the nature of the task that applied. One striking pattern is that the direction of modulation of cholinergic drugs on sensory cortex activity depends upon whether or not subjects attend to the stimulus. Thus Table 3A documents that when the stimulus was observed passively, or was irrelevant to task-set, cholinergic stimulation (with nicotine or cholinesterase inhibition) generally either elicited no effect (e.g. Jacobsen et al., 2002), or else suppressed sensory cortex – both in terms of overall activation strength and lateral spread – especially in early processing areas such as striate cortex (Silver et al., 2008). Cholinesterase inhibition also decreases visual cortex activations *independent* of task, suggesting a modulation of stimulus-driven processing alone (Bentley et al., 2004, 2008). Consistent with these results, the muscarinic receptor antagonist scopolamine results in enhanced visual cortex activations during paradigms involving passive exposure to visual stimulation (Mentis et al., 2001), or tasks involving auditory processing – i.e. when visual cortex is not stimulated via afferent pathways (Grasby et al., 1995; Bahro et al., 1999). Resting-state studies further support this general pattern with scopolamine tending to increase, but physostigmine decreasing, sensory cortex glucose consumption (Blin et al., 1994; Blin et al., 1997). Such findings suggest that stimulation of cholinergic receptors, especially muscarinic-type, can lead to net *suppression* of activity within early sensory cortical regions, for stimuli that are task-irrelevant.

In contrast, when the stimulus becomes task-relevant – either because of instructions for a sensory judgement (Table 3B) or to remember (Table 3C) – the opposite pattern is typically found. Thus, stimulus-evoked visual cortex activity is enhanced by cholinergic stimulation (e.g. Furey et al., 2000a), but suppressed by scopolamine (e.g. Antonova et al., 2010), in tasks requiring stimulus processing. In many cases, opposite types of sensory cortex modulation by the same cholinergic challenge, dependent upon task-related attention, can arise within the same experiment. For instance, physostigmine enhances the strength and spatial extent of activity in visual extrastriate cortex specifically during face-encoding (Furey et al., 2000b), and spatial attention (Bentley et al., 2004). By contrast, the same studies show that physostigmine induces negative modulation, or none, within the same sensory regions, during periods with similar stimulus properties but little or no stimulus-processing demands, or when the same stimulus property must now be ignored instead of attended (Bentley et al., 2003a).

A concordant pattern of effects is seen with nicotine. Nicotine induces higher visual cortex activity during a demanding visual maze task, rather than a visuomotor control task (Ghatan et al., 1998); while increasing auditory cortex activations more during an attention-demanding 2-back, rather than a 1-back, auditory working-memory paradigm (Jacobsen et al., 2004). In one study, where nicotine appeared to enhance visual extrastriate activity during both a working memory and a control task, it is noteworthy that even the control task required active attention to a series of rapidly presented visual digits (Lawrence et al., 2002). Nicotine also enhances occipital activity during visual target-detection tasks, particularly in trials preceded by spatially precise cues or using difficult, low-contrast targets (Hahn et al., 2007), or for invalidly cued targets (Thiel et al., 2005) – i.e. when attentional demands are relatively high in all cases. By contrast, nicotine decreases occipital activity on trials with spatially imprecise cues or high-contrast targets (Hahn et al., 2007), or with alerting but non-orienting cues (Thiel et al., 2005), in which cases there is relatively less demand for top-down processing. Consistently, cholinergic antagonism decreases occipital cortex activation selectively during trials requiring spatial orientation or with distractors (Thienel et al., 2009a,b).

A related observation is that subjects who show attentional impairments – e.g. through sleep-deprivation (Chuah and Chee, 2008), age (Freo et al., 2005; Ricciardi et al., 2009) or disease (Kumari et al., 2006; Goekoop et al., 2006; Bentley et al., 2008) – tend to exhibit a greater enhancement of (task-dependent) sensory cortex activity with cholinergic stimulation than seen in unimpaired subjects. This dependency upon state/trait is reflected in a greater performance response to cholinergic stimulation among poorly performing subjects (Kukolja et al., 2009; Bentley et al., 2009). Since less-able subjects are likely to experience greater difficulty than healthy subjects for a given task, and therefore require more attentional effort to achieve similar performance, these results parallel observations made above in healthy subjects (e.g. Jacobsen et al., 2004), that cholinergic stimulation increases sensory cortex activations selectively during attentional-demanding conditions.

#### 5.1.2 Interpretation

**Fig. 1:**
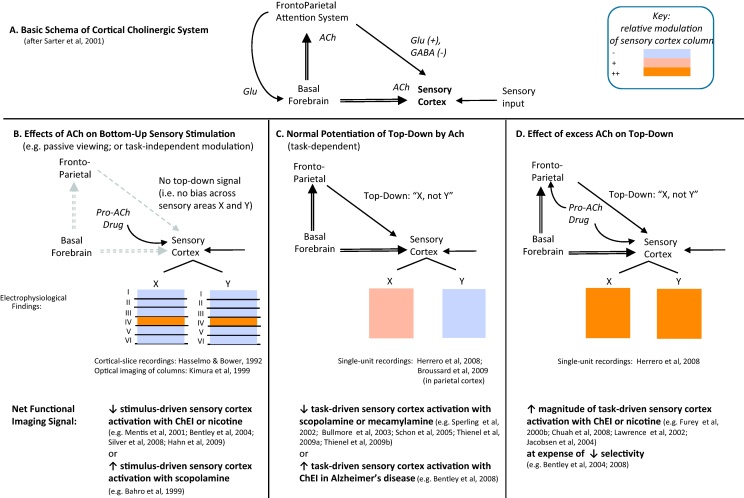
Model that links effects of acetylcholine on sensory cortex as appreciated from non-human electrophysiological studies, with effects observed in human functional imaging paradigms following systemic cholinergic stimulation or antagonism. (A) Schematic configuration of neocortical cholinergic system showing how sensory cortex receives cholinergic modulation both directly and indirectly via cholinergic modulation of frontoparietal processing. (B) Effects of ACh on the sensory cortical circuits are known for ex vivo slices, often with selective layer IV input activation, that are arguably most representative of passive-stimulation paradigms when top-down inputs are relatively low. In these situations, ACh application causes net neural suppression, that corresponds with findings from human functional imaging paradigms in which pro-cholinergic drugs result in sensory cortex suppression (or vice versa for scopolamine). (C) Task-driven selective activation of sensory or parietal cortex (e.g. as guided by the rule: X not Y) is found in non-human studies to be acetylcholine dependent. Correspondingly, cholinergic antagonists decrease task-relevant sensory cortex activations under attention-demanding conditions in human functional imaging studies. In Alzheimer's disease, where task-driven sensory cortex activations are abnormally low, and a cortical cholinergic deficit exists, administration of physostigmine increases selective sensory cortex activations. (D) Cholinergic hyperstimulation increases activity in both task-relevant and task-irrelevant units, in non-human electrophysiological recordings. If task-relevant units are already close to maximal firing, then this may lead to a greater increment in task-irrelevant units, explaining why in hypercholinergic states there may be an actual reduction in task-driven selective activation of sensory cortex, as seen in human functional imaging paradigms under ChEI or nicotine. *Abbreviations*: ACh, acetylcholine; Glu, glutamate; GABA, gamma-amino butyric acid; ChEI, cholinesterase inhibitor.

From the perspective of existing accounts of cholinergic impacts on sensory processing that recognise separable influences for bottom-up and top-down processes (Sarter et al., 2001) (Fig. 1A), the results of cholinergic functional imaging can be summarised as: (1) cholinergic stimulation typically *suppresses* (or cholinergic blockade enhances) net sensory activations under conditions in which *bottom-up* processing predominates – e.g. with passive or task-irrelevant, or task-independent, sensory stimulation; or alerting but non-orienting cues; while (2) cholinergic stimulation instead typically *enhances* (or cholinergic blockade decreases) net sensory cortical activations under conditions where *top-down* influences are strong, e.g. demanding perceptual discrimination, higher attentional load, orienting cues or deep memory encoding. Furthermore, cholinergic modulation of task-dependent sensory cortex activity correlates with drug effects on working memory (Furey et al., 2000b; Chuah and Chee, 2008) or short-term memory performance (Sperling et al., 2002; Schon et al., 2005; Bentley et al., 2009). This supports models where cholinergic influences on sensory cortices also influence attentional and memory functions (Hasselmo and McGaughy, 2004; Sarter et al., 2005).

Can we relate the profile of cholinergic modulation of sensory cortex activations, as found with PET or fMRI, to electrophysiological data? As discussed the effects of ACh stimulation as studied electrophysiologically are varied, with some potentiation of activity restricted to stimulus-driven units in layer IV, but the predominant modulation among other cortical layers, subserving feedback or lateral interactions, being suppressive (Hasselmo, 1995; Hasselmo and Cekic, 1996; Roberts et al., 2005). The net effects from such combined modulation, with qualitative differences between layers, is suggested by voltage-sensitive optical imaging which demonstrates that ACh generally suppresses overall strength and propagation of afferent-driven electrical activity within and between columns of cat visual cortex (Kimura et al., 1999) (Fig. 1B). From a functional perspective, widespread neural suppression may ‘reset’ sensory processing (Gulledge et al., 2007), thereby heightening signal-to-noise ratio specifically for sensory, i.e. thalamocortical inputs (Sato et al., 1987b), while reducing lateral or feedback influences (Gil et al., 1997; Roberts et al., 2005). By comparison with functional imaging data, it is apparent that pro-cholinergic drugs are also often associated with reduced sensory activation magnitude (Bentley et al., 2004) or spread (Silver et al., 2008) (or vice versa for anti-cholinergics, e.g. Bahro et al., 1999), specifically during low-attention or passive stimulation paradigms.

Following the schema of Sarter et al. (2001), and bearing in mind that most electrophysiological studies measure stimulus-evoked responses divorced from top-down inputs, the functional neuroimaging findings that pro-cholinergic drugs decrease stimulus-evoked sensory cortex activations under low-attention conditions correspond to electrophysiological findings of ACh-induced suppression of *overall* activity in sensory cortex – i.e. when spatially summing over all layers of a cortical column. (Note, how this is distinct from the earlier point that the majority of sensory neurons show facilitation in response to ACh.) However, although the net signal is less than normal, we know that in this situation (e.g. Kimura et al., 1999) cholinergic stimulation actually increases afferent activity within layer IV while reducing interference from lateral or top-down inputs, and so, this functional imaging signature of sensory cortex hypoactivation may be considered to be a marker of enhanced bottom-up processing. Behaviourally this is supported by human fMRI studies showing that cholinesterase inhibition speeds reaction times in a visual-stimulus detection task, while suppressing striate cortex activations, independently of task requirements in both cases (Bentley et al., 2004). Conversely, anti-cholinergics are associated with slower target detection, and increased occipital cortex activations, specifically in trials without spatial cues or target-conflict – i.e. when top-down requirements are less (Thienel et al., 2009b).

If *decreases* in sensory cortex activation induced by cholinergic stimulatory drugs reflect neural suppression of lateral or feedback influences as seen following ACh application to cortical slices, then what neurophysiological events do pro-cholinergic drug-induced *increases* in sensory activation relate to, as are generally found in high-attention conditions within neuroimaging paradigms? To recap, a critical role for the cholinergic system is to maintain sensory processing in the face of performance challenges such as distractors (Sarter et al., 2006). Thus we would expect ACh to potentiate neural correlates of selective attention, in which sensory processing is biased towards task-relevant stimulus features, and away from task-irrelevant ones. In keeping with this, two recent studies in awake monkeys and rats respectively, indicate that cholinergic input to sensory (Herrero et al., 2008) and parietal (Broussard et al., 2009) cortices can potentiate neural correlates of selective attention by disproportionately increasing weighting of task-relevant versus task-irrelevant inputs (Fig. 1C). However, of relevance here, is that ACh application also increased the *overall* level of visual neural activity, both in cells coding for task-relevant and task-irrelevant locations, specifically during target detection. Accordingly, with attention-demanding, relative to baseline, conditions, as listed in Table 3B and C, we might expect pro-cholinergic treatments to enhance stimulus-evoked responses at the spatial scale of fMRI or PET, that integrate activity over thousands of such units (potentially including both task-relevant and task-irrelevant). The potential implications for the *differential* activation of task-relevant versus task-irrelevant sensory units are discussed in Section 5.3.

Combining neurophysiological accounts of cholinergic modulation on bottom-up (e.g. Hasselmo and McGaughy, 2004) and top-down (e.g. Herrero et al., 2008) processes within sensory cortices, we propose an account that accommodates the attention-dependent profile of cholinergic impact on sensory activations studied with neuroimaging (see Fig. 1B–D). Whenever a stimulus is presented, regardless of task, we expect cholinergic stimulation to facilitate bottom-up circuitry, while reducing feedback and horizontal influences – the net metabolic signature of which may be decreased sensory cortex activation (e.g. Kimura et al., 1999) (Fig. 1B). Conversely, in a subset of sensory paradigms, in which attention is focused towards the stimulus, top-down glutamatergic-mediated signals will enhance activity in selected, task-relevant sensory regions. Knowing that ACh potentiates neural activation of task-relevant, relative to task-irrelevant, sensory cortical regions (Herrero et al., 2008), we might expect human functional imaging studies to reveal a reduction in task-driven sensory cortex activity following cholinergic antagonism, as is seen (Fig. 1C). Furthermore, the electrophysiological finding that both task-relevant and task-*irrelevant* sensory units increase in firing frequency with cholinergic stimulation (Herrero et al., 2008) provides a potential explanation as to why further increases in both magnitude and spatial extent of task-driven sensory cortex activations can be observed in healthy subjects administered a cholinesterase inhibitor or nicotine (Fig. 1D). The hypothesis that in these human paradigms, pro-cholinergic drugs exaggerate top-down amplification of sensory signalling is supported by findings that such sensory modulations are more apparent in subjects with poorer baseline performance (e.g. Chuah and Chee, 2008; Bentley et al., 2008) – for whom it is plausible that a greater top-down ‘attentional effort’ is operative in order to sustain error-free performance (Sarter et al., 2006). Alternatively, impaired subjects may start off having lower tonic acetylcholine, and task-related activation, levels than normal, allowing for a greater dynamic range of responses secondary to pro-cholinergic therapies than seen in healthy subjects (see also Section 8).

### 5.2 Anatomical variations of sensory cortex modulations

Several neuroimaging experiments probing *visual* cortex reveal an anterior – posterior gradient of cholinergic modulation, suggesting differential influences between early versus higher visual processing. For example, physostigmine decreases stimulus-induced visual striate cortex activations (Silver et al., 2008), and can do so regardless of task (Bentley et al., 2004); but increases them in higher extrastriate visual regions in a task-specific manner (Furey et al., 2000a; Bentley et al., 2004). Similarly, during visual tasks, nicotine decreases posterior visual cortical activations while increasing those in more anterior visual regions (Thiel et al., 2005; Hahn et al., 2009). Furthermore, scopolamine decreases activations in extrastriate visual cortex specifically during face–name learning, whereas no modulation is observed in striate cortex (Sperling et al., 2002; Thiel et al., 2001). These findings suggest that primary visual cortex may be less susceptible to cholinergic modulation, especially in its interaction with top-down factors. One way by which such anatomical-specificity of ACh effects may occur is through receptor segregation – for example, a preferential expression of muscarinic receptors in V2 relative to V1 cortex parallels a spatial gradient in attentional modulation (Disney et al., 2006).

A further consistent anatomical division by which visual regions differ according to cholinergic response arises between ventromedial and posterolateral visual regions, with the former showing increases, and the latter decreases, in activity following cholinergic stimulation. Across three separate paradigms, involving either active or passive viewing, physostigmine increases stimulus-induced, ventromedial extrastriate activations (including fusiform gyrus), at the same time as decreasing activations in posterolateral occipital regions (Furey et al., 2000b, 2008a; Ricciardi et al., 2009; Bentley et al., 2003a; Mentis et al., 2001). Results from studies using nicotine (Thiel et al., 2005; Hahn et al., 2007) or varenicline (Loughead et al., 2011) as the cholinergic stimulant concord with this pattern. Conversely, muscarinic blockade results in activation decreases in fusiform cortex (Thiel et al., 2002c; Sperling et al., 2002; Schon et al., 2005; Rosier et al., 1999) or medial occipital cortex (Dumas et al., 2010), but activation increases in lateral occipital cortices (Grasby et al., 1995; Bahro et al., 1999; Mentis et al., 2001; Thienel et al., 2009b; Dumas et al., 2010).

Cholinergic-induced enhancement of inferior-medial temporal cortex might relate to this region's critical role in stimulus encoding for later memory. Since activation in inferior temporal cortex can index subsequent memory (Grady et al., 1998), cholinergic-induced enhancements here may reflect facilitation of encoding (Bentley et al., 2009), possibly due to processes such as persistent-spiking (Klink and Alonso, 1997; Hasselmo and Stern, 2006). This might explain why cholinergic modulation of medial, but not lateral, occipital regions increases with temporal delay between encoding and subsequent memory testing (Furey et al., 2008a). Conversely, lateral occipital cortex, that is heavily influenced by top-down and lateral connections (Vinberg and Grill-Spector, 2008), might be expected to show depressed activity following cholinergic stimulation, given that ACh generally inhibits intracortical transmission (Kimura et al., 1999; Roberts et al., 2005). An anatomical basis for a medial–lateral occipital dissociation of cholinergic responsivity is hinted at by findings that cholinergic fibres to human occipital cortex segregate into medial and lateral tracts (Selden et al., 1998).

### 5.3 Modulations of attentional effects within sensory cortex

Neocortical cholinergic afferents play a key role in selective attention (Sarter et al., 2006), with evidence from single-unit rat and monkey studies of ACh potentiating attentional modulation of visual (Herrero et al., 2008) and parietal (Broussard et al., 2009) responses. One might therefore expect pro-cholinergic drugs to enhance neural correlates of selective attention in sensory cortices as measured by functional neuroimaging, e.g. differential activation of retinotopic visual cortex as a function of spatial cueing (Martinez et al., 2001). It was therefore unexpected when several functional imaging paradigms reported that cholinesterase inhibition appeared to reduce top-down, selective effects in sensory cortices. This was seen for both spatial attention (Bentley et al., 2003a, 2004) and depth-of-processing (Bentley et al., 2008) visual tasks, in which physostigmine actually reduced task-driven (as opposed to stimulus-driven) modulation of extrastriate visual cortices. Similarly, in a fear-conditioning paradigm, physostigmine reduced the differential activation of auditory cortex to a conditioned stimulus (i.e. previously paired with a shock) relative to a non-conditioned stimulus (i.e. no shock association) (Thiel et al., 2002b). Physostigmine has also been shown to increase the spatial extent of visual cortex activations during a face working memory task, implying a reduction in task-driven visual-selectivity (Furey et al., 2000a).

To reconcile this set of findings with those described earlier (Section 5.1) – that pro-cholinergic drugs generally elevate functional activations during attention-demanding tasks – these experiments also showed that a main reason for such decreases in attentional selectivity is because of a disproportionate increase in sensory activity for task-irrelevant (or non-conditioned), rather than a decrease for task-relevant (or conditioned) stimuli (Bentley et al., 2004, 2008; Thiel et al., 2002b). Moreover, behavioural data acquired during scanning show that enhancement of unattended stimulus processing associated with a hypercholinergic state has functional consequences. For example, enhanced activation of visual cortex contralateral to invalidly cued (in this sense, unattended) targets, due to physostigmine, correlates with behavioural speeding of performance for them (Bentley et al., 2004). Furthermore, high-ACh states can enhance behavioural (Holley et al., 1995) and autonomic (Quigley et al., 1994) responses to irrelevant or low salience (Furey et al., 2008b) stimuli. Thus, by heightening activity in sensory regions away from those favoured by top-down commands, a hypercholinergic state can increase detectability of unexpected or invalidly cued signals. This fits a computational model outlined in Section 2.2, in which heightened cortical ACh levels serve to reduce endogenous weighting of inputs under conditions of high uncertainty (Yu and Dayan, 2005).

Nicotine may induce similar influences on selective attention as cholinesterase inhibition. One consistently observed behavioural effect is that nicotine reduces the penalty incurred by invalid attentional cueing (Witte et al., 1997; Thiel et al., 2005), suggesting that it can reduce endogenous weighting, paralleling effects of physostigmine described above. Furthermore, nicotine reduces correlation between occipital deactivations and increasing spatial precision of a cue in a target-detection task, suggesting that it enhances activity in task-irrelevant retinotopic areas (Hahn et al., 2007). However, nicotine does not consistently modulate cue-driven selectivity in visual cortex (Thiel and Fink, 2008), suggesting that more regionally abundant muscarinic receptors (Paterson and Nordberg, 2000; Zilles et al., 2002) may account for the full profile seen with physostigmine (Bentley et al., 2004).

Does evidence from other techniques, including invasive cellular recordings, also indicate a hypercholinergic state decreases attention-related selectivity in sensory cortex? As mentioned, local ACh application in visual cortex can increase the difference in firing rates between cells coding for task-relevant versus task-irrelevant locations (Herrero et al., 2008). However, the same study also found that ACh increased the *overall* firing rate; and, moreover, in some neurons ACh increased it disproportionately more for stimulus-attribute values (e.g. bar length) that were non-optimal for the neuron's usual tuning preferences. Other studies have noted ACh-induced reductions in selectivity to stimulus features (Zinke et al., 2006) or spatial coding (Kuo et al., 2009), at the same time as enhancing overall activity. This concords with functional imaging findings of enhanced sensory cortex activation levels following cholinergic stimulation, concomitantly with reduced selectivity (Fig. 1).

Conceivably, under hypercholinergic conditions – i.e. those achievable pharmacologically, but not encountered under ‘normal’ physiological states – weak top down signals are boosted more than strong ones, because the latter have already reached a ceiling. This might explain why some cholinergic-functional imaging results seem maladaptive in the sense that they apparently favour task-irrelevant (e.g. invalidly cued) over task-relevant (e.g. validly cued) stimulus processing. Apparent support for this interpretation comes from an animal model of anxiety and psychosis (Berntson et al., 1998), showing that excessive ACh neurotransmission produces a hypervigilant state – including heightened sensitivity to distractor, irrelevant stimuli. Additionally, acute nicotine challenges in non-smokers can result in hyperarousal and anxiety (Kobiella et al., 2011).

## 6 Functional neuroimaging: attentional modulations in frontoparietal regions

**Fig. 2:**
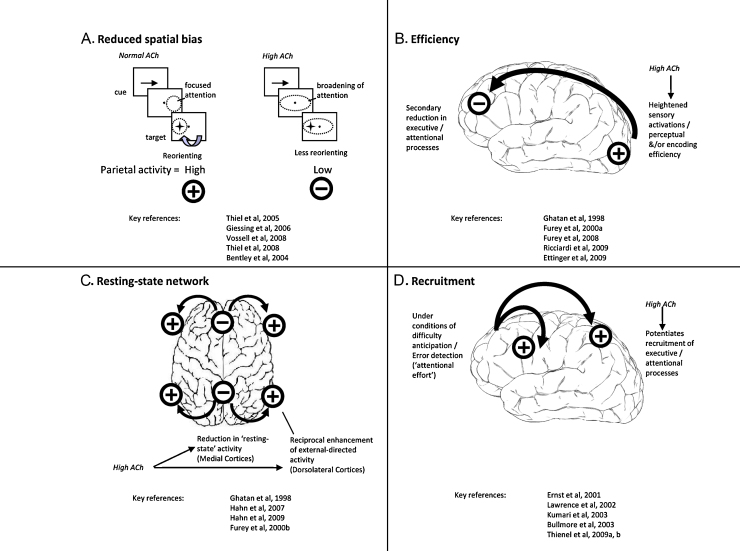
Explanations for modulations of frontoparietal activations in cholinergic-functional imaging studies. (A) Decreases in parietal activation during re-orienting trials secondary to pro-cholinergic drugs (especially nicotine) may occur indirectly because of a hypercholinergic-induced reduction in spatial biasing. (B) Decreases in frontoparietal activation secondary to pro-cholinergic drugs may also be secondary to direct effects of cholinergic stimulation in sensory cortical regions, which result in heightened efficiency, and thus less ongoing need for executive control. (C) Decreases in medial frontal–parietal activations secondary to pro-cholinergic drugs overlap with a recognised resting-state network, which together with drug-induced reciprocal increases in activity in dorsolateral regions, suggests a state change from internally focused feedback-predominant mode to externally directed feedforward mode. (D) Increases in frontoparietal activations secondary to pro-cholinergic drugs, specifically during demanding task conditions, and sometimes with performance improvement, suggest recruitment of additional executive-attentional processes. Note that thick arrows are intended to show possible order by which processes are modulated, and not anatomical connectivity.

The next four subsections (Sections 6.1–6.4) interpret cholinergic neuromodulations of frontoparietal activity as revealed by human functional imaging studies (Table 4) according to one of four general schemes. In the first three, the discussion focuses on those results where pro-cholinergic drugs suppress task-specific frontoparietal activity (or, consistent with this, where anti-cholinergics increase activity), while the fourth attempts to explain why in other circumstances, the opposite profile is seen: i.e. frontoparietal *hyper*-activation secondary to pro-cholinergic therapies (or suppression by cholinergic blockade). These accounts (see also Fig. 2A–D) can be summarised as follows: (1) pro-cholinergic reductions in parietal activity are associated with reduced attentional orienting; (2) pro-cholinergic reductions in frontal activity may occur due to enhanced sensory processing, or via other efficiency-enhancing mechanisms, thereby requiring less ‘attentional effort’; (3) pro-cholinergic reductions in activity of a predominantly medially located, resting-state network suggest a shift from internal to external (i.e. stimulus) processing; and (4) pro-cholinergic increases in activity, especially of a dorsolateral frontoparietal network, may reflect increased recruitment of attentional-executive processes. To extend comments made under Sections 3.2–3.3, we emphasise here the fact that the same frontoparietal regions show either increases or decreases in activity secondary to the same drugs, in the same subjects, albeit under different cognitive conditions, strongly argues against general modulation of vascular responses, but rather invites an interpretation in terms of neuropsychological interactions. The interpretations that follow are intended to draw together the most consistent findings within Table 2, and are not intended to be exhaustive.

### 6.1 Effects on top-down control of attentional orienting

If cholinergic stimulation reduces top-down modulation of sensory cortices (see Section 5.3), then we might expect the same drugs to modulate those frontoparietal regions – notably including right parietal cortex – believed to exert top-down control of attention (e.g. Yantis et al., 2002). Consistent with this, both physostigmine (Bentley et al., 2008) and nicotine (Rose et al., 2010), reduce parietal activity during selective attention paradigms, while also causing a reduction in task-driven, differential sensory cortex activation (Bentley et al., 2004).

A related observation originates from studies employing a version of the Posner spatial cueing task. In those studies, nicotine consistently decreases inferior parietal cortex activations specifically during invalidly cued trials, when there is a need for reorienting away from a cued location (Thiel et al., 2005; Thiel and Fink, 2007, 2008) (Fig. 2A). Since nicotine also decreases the performance-cost of invalid cues (Phillips et al., 2000; Thiel et al., 2005), this decreased parietal activation during invalid trials seems not to reflect impaired reorienting, but rather a processing benefit for the invalidly cued trials. Further variations of this paradigm reveal that nicotine-induced decreases in parietal responses to invalid cues are diminished when cue-derived expectation is reduced, i.e. by decreasing the relative probability of valid trials in which targets appear at the cued location (Vossel et al., 2006, 2008; Giessing et al., 2006). Taken together, these data suggest that nicotine decreases cue-elicited spatial biasing – thereby secondarily reducing parietal-mediated reorientation to targets at uncued location, because participants are already less committed to the cued location when the uncued target appears. The fact that performance may be enhanced by nicotine on invalid trials is consistent with cholinergic stimulation favouring bottom-up over top-down processing. This squares with the point made earlier (Section 5.3) that physostigmine reduces top-down driven selective activation of sensory cortices, while increasing sensory responses in general. Moreover, cholinergic effects on parietal-mediated reorienting for invalidly cued targets satisfies for invalidly cued targets satisfies a key prediction of a model proposing that ACh reduces inference-driven biasing of sensory cortex, to the benefit of stimulus-driven signalling during periods of ‘expected’ uncertainty (Yu and Dayan, 2005). However, nicotine itself has not been found to reduce attentional modulation of sensory cortex, perhaps suggesting that muscarinic receptors are essential for the latter effects (see Thiel and Fink, 2008).

Other patterns of frontoparietal modulation by cholinergic drugs support such an account of acetylcholine reducing top-down attention. First, nicotine reduces anterior cingulate, as well as parietal cortex, activity during invalid trials, coincident with speeding and reduced response variability (Vossel et al., 2008). Given that anterior cingulate acts as a source of attentional control (Sarter et al., 2006), a nicotinic-induced reduction in its activity might reflect reduced ‘attentional effort’ and/or error detection, on invalidly cued trials, due to less of a top-down bias towards the cued location. Second, nicotine decreases right angular gyrus activations during uncued relative to cued (i.e. ‘alerting’) trials (Thiel et al., 2005; Thiel and Fink, 2007). Since this region appears to mediate reorienting to unattended stimuli (Yantis et al., 2002), this suggests that nicotine reduces the ‘surprise’ element of uncued stimuli, possibly by heightening vigilance (Wesnes and Warburton, 1984), and thus reducing the subsequent need to reorient. Finally, and mirroring pro-cholinergic reductions in parietal activity, anti-muscarinic or anti-nicotinic drugs (i.e. scopolamine or mecamylamine) increase parietal activity during a visual attention task, with associated impaired performance (Thienel et al., 2009a,b). Since these drug-induced hyperactivations occurred selectively with target–distractor conflict, when parietal activity might reflect attentional refocusing (Corbetta et al., 2000), and given that performance was most impaired by these drugs during conflict trials, the parietal hyperactivations here may be because anticholinergics decreased selective attention to the cued target location prior to target appearance. Thus both hypocholinergic and hypercholinergic states can be associated with parietal and performance modulations that suggest impairment in top-down processing (see also Section 8).

In the absence of reliable cues, target-associated activations within inferior parietal or adjacent supramodal superior temporal gyrus may reflect processing within a stimulus-driven, bottom-up ‘interrupt’ system (Corbetta et al., 2000), rather than top-down orienting. If cholinergic stimulation favours bottom-up over top-down processing, then we might expect pro-cholinergic therapies to increase such activations – which is indeed what is found. Hence, superior temporal gyrus activity increases with nicotine in uncued trials, but decreases in cued trials, resulting in a ‘levelling out’ of responses (Thiel and Fink, 2007). Furthermore, with poorly predictive cues, nicotine increases target-induced parietal activations (Vossel et al., 2008; Giessing et al., 2006), that may reflect nicotine potentiating bottom-up processing of unexpected stimuli, or registering of uncertainty (Yu and Dayan, 2005). This is compatible with nicotine speeding responses to highly salient stimuli selectively (Rycroft et al., 2005); and parietal modulation by nicotine correlating with performance improvements selectively for high-, rather than low-, intensity targets (Hahn et al., 2007). Taken together with the observations made at the start of this subsection, these results indicate that cholinergic stimulation may suppress top-down enhancement of subtle inputs (i.e. orienting), by favouring a state in which bottom-up inputs compete for attention by virtue of their salience. Note also how this molds with conceptualisations of cholinergic modulations of sensory processing (see Section 2.1) – by which acetylcholine enhances sensory unit firing proportionately to the degree of afferent input (Krnjević and Phillis, 1963), and favours feedforward over feedback processing (Hasselmo and McGaughy, 2004).

### 6.2 Efficiency of cortical processing

When a drug reduces task-related activity, and at the same time, improves performance, one parsimonious account is to suggest that the drug enhances cortical processing efficiency. This is analogous to non-pharmacological functional imaging paradigms, where correlations between enhanced performance and reductions in prefrontal activations have been interpreted in terms of efficiency (Rypma et al., 2006), presumably because of reduced processing times; smaller volumes of active cortex, fewer numbers of locally recruited neurons, reduced firing rates, etc., which together result in less metabolic demands. Regional hypoactivation may reflect improved processing efficiency within the region itself; in remote region(s) that provide input to the modulated area; or in the interconnections between them.

Numerous examples exist whereby pro-cholinergic drugs improve performance while decreasing frontoparietal activation (Table 4). For example, physostigmine-induced reductions in dorsal prefrontal cortex activity, during encoding and maintenance-phases of a working memory task (Furey et al., 2000a), have been interpreted in terms of reduced task effort, on account of a correlation between these imaging effects and drug-induced speeding of responses (Furey et al., 1997). One explanation (Fig. 2B) is that physostigmine produces a more robust neural representation of studied stimuli – indexed by enhanced responses in visual extrastriate regions during encoding (Furey et al., 2000a) – thereby necessitating less prefrontal, executive-related activity during a subsequent working memory delay period. Since such drug effects on BOLD responses and performance are more marked at longer memory delays (Furey et al., 2008a; Ricciardi et al., 2009), the benefit appears to be specific for memory processes, e.g. by enhancing stimulus-specific persistent-spiking in higher sensory-perirhinal cortices (Klink and Alonso, 1997; Hasselmo and Stern, 2006), rather than being directly related to stimulus processing, retrieval or motor response.

The idea that drug-induced facilitation of sensory processing, or encoding, secondarily decreases prefrontal activations is complementary to findings from non-pharmacological functional imaging studies that prefrontal activation scales with sensory processing demands (Grady et al., 1996). Moreover, the general observation that pro-cholinergic manipulations lead to reciprocal modulations between frontoparietal and sensory regions is supported by studies showing the opposite profile with cholinergic antagonists. Hence scopolamine decreases fusiform cortex activations, at the same time as increasing parietal (and thalamic) activations, during the recollection stage of a visual memory task (Rosier et al., 1999). Similarly, either scopolamine or mecamylamine decrease visual cortex activation, while increasing frontal activations (Dumas et al., 2010), suggesting greater executive processing for the same performance, and paralleling a recognised ‘Posterior-to-Anterior Shift in Aging’ activation profile (Davis et al., 2008).

Diminutions in prefrontal activity, in association with improved performance, are also found with nicotine (Hahn et al., 2009; Ettinger et al., 2009). Once again, such nicotine-induced reductions in prefrontal activity during a perceptual task are associated with increased posterior cortical activations (Ghatan et al., 1998), suggesting that nicotine may primarily enhance sensory processing efficiency. In the case of nicotine, these findings may also reflect direct effects within prefrontal cortex itself, by, for example, nicotine-induced facilitation of presynaptic neurotransmitter release, without an increase in presynaptic electrical activity (Vidal and Changeux, 1993; Lambe et al., 2003; Wonnacott et al., 2006). Furthermore in some subject groups, e.g. as characterised by dopamine receptor genetic polymorphisms, nicotine may actually worsen performance while being associated with *hyper*activations in relevant processing regions, e.g. in left anterior insula – a critical node within an ‘articulatory-loop’ during an auditory working memory task (Jacobsen et al., 2006), suggesting a worsening of efficiency.

An additional explanation for nicotine-associated modulations in frontal activity, and improved performance, is that it induces a positive emotional state, or arousal, which may be indirect drivers of efficiency (Eysenck et al., 2007; Foster et al., 2008). This is especially relevant in the context of studies scanning smokers, in whom nicotine serves to relieve withdrawal symptoms. Typically, the comparator of these experiments is placebo, which in this set of subjects may actually signify the development of negative symptoms such as craving. Thus, in these situations, nicotine-induced reductions in prefrontal activity, associated with improved performance (Ernst et al., 2001a; Xu et al., 2007; Azizian et al., 2010), may actually signify the removal of distracting physical and emotional symptoms, thereby necessitating less attentional control.

Do reduced activations in frontoparietal cortex secondary to cholinergic stimulation reflect enhanced processing efficiency in general, or are they functionally specific? On the one hand, nicotine-induced deactivations of frontoparietal cortices (and thalamus) correlate with response speeding, yet do not interact with cue predictivity in a spatial attention task (Hahn et al., 2007, 2009). This suggests that nicotine may exert a general preparatory or alerting effect in frontoparietal regions, rather than interacting with spatial orienting per se. However, in other cases, pro-cholinergic treatments induce modulations that are context-specific, e.g. hypoactivation of frontoparietal cortex during spatial attention, but not spatial working memory (Bentley et al., 2004); with invalidly, rather than validly, cued targets (Vossel et al., 2008) or with incongruent, rather than congruent, Stroop targets (Xu et al., 2007). Moreover, the profile of these pharmacological interactions suggests that they do not merely arise from differences in the degree of task-induced cortical activation in the absence of drug (e.g. due to proportionate scaling), but possibly reflect differences in local processing or inputs between differing cognitive contexts.

One difficulty in interpreting drug-induced reductions in frontoparietal activations, in association with performance improvements, relates to the issue of reaction time confounding. By this argument, a shortening of reaction times implies that the total amount of task-specific processing between stimulus and response is less, which in itself would give rise to smaller hemodynamic responses. Hence drug-induced cortical hypoactivations may be a *result* of enhanced efficiency – possibly due to effects in other brain regions – rather than being the *cause* of it. For this reason, it is useful to test whether pharmacological neuromodulations are still observed after partialling out reaction time effects (e.g. Bentley et al., 2008), in which case such confounds are less significant (although, in such cases, the neural modulations observed are arguably less relevant as explanations for the performance change).

A further caveat to an efficiency account of cholinergic frontoparietal modulations is that it clearly cannot account for all results, as seen by the number of contradictory examples in Table 4. For example, cholinergic antagonists, like pro-cholinergic drugs, also reduce task-associated frontoparietal activations, but are associated with performance *impairments*, e.g. with visual discrimination (Thienel et al., 2009a,b), or memory tasks (Sperling et al., 2002; Bullmore et al., 2003; Craig et al., 2009). Alternatively, pro-cholinergic drugs can improve performance while *increasing* (e.g. Chuah and Chee, 2008), or while having no detectable effect (Ettinger et al., 2009; Sutherland et al., 2011), on frontoparietal activity. In some cases even, nicotine may reduce prefrontal activity, while being accompanied by *performance impairment* (Jacobsen et al., 2004, 2006). Such findings necessarily demand additional interpretations, such as supposing that some frontoparietal activations are critical for task performance (and hence positively correlate with performance), rather than reflecting extraneous ‘effort’ (which negatively correlate with performance); or in the case of bi-directional nicotine effects by supposing that whether efficiency is increased or decreased depends upon baseline cholinergic tone, or performance – i.e. that responses follow an inverted-U shaped profile (discussed further in Section 8).

### 6.3 Default network

Many fronto-parietal–temporal regions whose activity is suppressed by pro-cholinergic treatments (notably including nicotine) are either medially located (e.g. cingulate, precuneus, and parahippocampal gyri), or involve superior–middle temporal, and angular gyri (Ghatan et al., 1998; Bentley et al., 2004; Hahn et al., 2007, 2009; Ettinger et al., 2009; Azizian et al., 2010; Loughead et al., 2011). These regions overlap with the so-called ‘default’ or ‘resting-state’ network (Raichle and Snyder, 2007), and as such suggest another mechanism by which the cholinergic system and cholinergic drugs may act. Cholinergic stimulation typically exaggerates deactivations within these regions, seen without drug during attention-demanding tasks, while not affecting activity at rest. At the same time, many of these studies also show that cholinergic stimulation increases task-related activity in dorsolateral frontoparietal or posterior regions, suggesting a reciprocal shift in the balance of processing or activation between ‘resting-state’ and ‘attentional-sensory’ cortices (Fig. 2C). Conversely, hyperactivations are seen in medial frontoparietal regions with nicotine in the resting state, or with low-attention tasks (Stein et al., 1998; Lawrence et al., 2002; Kumari et al., 2003); or with anti-cholinergics during taxing tasks (Dumas et al., 2010; Antonova et al., 2010).

Given the similarity between nicotinic-mediated, task-related hypoactivations and the ‘resting-state’ network, it has been suggested that this pattern of pharmacological neuromodulation may represent a switch in processing from an internally focused state to one where sensory processing is required (Hahn et al., 2007) (see Fig. 2C). The fact that such drug-induced hypoactivations occur independently of the level or type of attention (Hahn et al., 2009) implies that cholinergic modulation may act to focus attention towards any externally specified task. Furthermore, positive correlations of nicotine-induced deactivations with performance appear in keeping with the idea that trial-to-trial performance depends upon the efficiency with which the resting-state network can be deactivated, possibly because of a reciprocal enhancement of task-relevant processing (Polli et al., 2005).

A cholinergic-mediated transition from a resting-state, internally focused network to one favouring processing of external stimuli would fit with the recognised capacity for acetylcholine to switch cortical dynamics from a cortico-cortical, or feedback state, to one that favours thalamocortical, or input-driven, signalling (Gil et al., 1997; Hasselmo and McGaughy, 2004). Hence to extend our earlier discussion of sensory cortex effects (Section 5.1), the neuroimaging signature of cholinergic-enhancement of bottom-up processing may include both sensory cortex suppression (e.g. Bentley et al., 2004; Silver et al., 2008), and enhanced deactivations of a resting-state default network.

As a caveat, it should be considered whether nicotine-induced response speeding may itself have led to some of the relevant deactivations (e.g. Herath et al., 2002), although BOLD-behavioural correlations were found only under certain conditions, or restricted to the thalamus (Hahn et al., 2007, 2009). Furthermore, nicotine-induced *hype*ractivations of anterior cingulate can be associated with positive performance effects (Ernst et al., 2001a; Kumari et al., 2003), while cholinergic blockade is associated both with hypoactivations in similar regions and with performance impairment (Grasby et al., 1995; Thienel et al., 2009a,b), indicating that not all medial cortical regions respond homogeneously. Furthermore, nicotine-induced hypoactivations of medial prefrontal regions may occur specifically in conflict scenarios (Hahn et al., 2007; Vossel et al., 2008), while speeding responses (Hasenfratz and Bättig, 1992), suggesting a more selective interpretation.

### 6.4 Recruitment of cortical processes

Certain studies show that pro-cholinergic drugs *increase* activation in frontoparietal regions (e.g. Ernst et al., 2001a; Lawrence et al., 2002; Bentley et al., 2004; Thiel et al., 2005), in contrast to the profile of nicotinic or physostigmine-induced deactivations discussed in Sections 6.1–6.3. Many of these drug-induced increases correlate positively with performance improvements. Consistently, multiple studies demonstrate that cholinergic blockade engenders task-related, frontoparietal hypoactivations, concomitant with performance decrements (e.g. Cohen et al., 1994; Bullmore et al., 2003; Thienel et al., 2009a,b). One factor that can account for the discrepancy of these findings with the pro-cholinergic associated hypoactivations described earlier (or anti-cholinergic associated hyperactivations) is anatomical. Pro-cholinergic *deactivations* tend to occur predominantly in *medial* prefrontal–parietal locations; whereas increased *activations* induced by cholinergic stimulants are often in *dorsolateral* frontoparietal cortices (Fig. 2C; as discussed in Section 6.3). This supports the suggestion that ACh biases processing away from an internally directed resting-state, and towards active processing of the environment, or task-engagement (Hahn et al., 2007).

A different sort of explanation is required to account for situations in which the *same* (usually dorsolateral) frontoparietal regions show either increases or decreases in neural activation, in response to a given cholinergic drug, depending upon condition. One pattern is that increases in frontoparietal activity secondary to pro-cholinergic drugs often occur specifically during the most challenging stimulus or task conditions (e.g. Lawrence et al., 2002; Bentley et al., 2004; Hahn et al., 2007, 2009; Hong et al., 2009; Loughead et al., 2010), often with associated performance improvements. Conversely, anti-cholinergics reduce activations in these same regions and impair performance during the most attention-taxing conditions (Bullmore et al., 2003; Bozzali et al., 2006; Thienel et al., 2009a). Furthermore, it should be noted that in most studies testing nicotine or varenicline, the comparator (placebo) is likely to reflect a period of abstinence, when subjects may experience adverse symptoms; and so this context may accentuate any interaction of drug with difficulty or attentional effort, relative to non-smokers. This may account for the fact that nicotinic stimulation increases frontoparietal activations selectively during the most difficult (3-back) working memory condition in smokers (Loughead et al., 2010), but not non-smokers (Kumari et al., 2003).

One interpretation of these findings is that ACh mediates recruitment of performance-dependent frontoparietal activity selectively when resources are pushed to near-maximum use, with the effect of enhancing rate-limiting step processing (Fig. 2D). In support of this, prefrontal cholinergic inputs are essential for increases in prefrontal activity that occur with distraction during a stimulus-detection task (Gill et al., 2000), while lesions to this input impair performance specifically in this demanding context. Furthermore, a principal set of triggers for cholinergic release is performance challenges – i.e. when ‘attentional effort’ is required, with prefrontal cortex then both triggering and receiving cholinergic stimulation (Sarter et al., 2006). It is possible that pro-cholinergic enhancements of prefrontal activity, seen in human functional imaging studies specifically during attention-demanding conditions, reflect accentuated persistent spiking secondary to cholinergic transients, that facilitate cue detection and subsequent behavioural priming (Parikh et al., 2008; Hasselmo and Sarter, 2011). Moreover, evidence from non-human studies that a prefrontal–cholinergic basal forebrain loop becomes co-activated, at the same as potentiation of sensory cortex (Golmayo et al., 2003), is supported by human neuroimaging studies demonstrating positive three-way correlations between cholinergic drug modulation of frontoparietal cortices, visual cortices and accuracy (Chuah and Chee, 2008; Bentley et al., 2009; Thienel et al., 2009a,b; Sweet et al., 2010).

Other examples of pro-cholinergic frontoparietal hyperactivations can also be interpreted in terms of processing recruitment. For example, activation of prefrontal cortex processing by nicotine is seen selectively with emotionally negative, rather than positive, stimuli, possibly due to the former evoking greater ‘bottom up’ attention (Kobiella et al., 2011). Additionally, frontoparietal hyperactivations due to nicotine, seen during periods of low cue predictivity (Thiel et al., 2005; Giessing et al., 2006; Vossel et al., 2008), and interpreted earlier as reduced attentional reorienting (Section 6.1), could alternatively be interpreted as cholinergic-recruitment of vigilance-related processing during periods of poor target predictability, or heightened task difficulty. Similarly, in an attentional paradigm showing predominantly nicotine-induced pan-cortical deactivations, it was noted that the few examples of drug-associated frontoparietal *hyperactivations* occurred in the most taxing task condition, viz. invalidly cued low-intensity targets (Hahn et al., 2007). However, there was no behavioural correlation with these neural modulations, and the same study also showed that nicotine-induced prefrontal hyperactivations correlated with nicotine-induced performance *impairments*, during a relatively easy task condition, viz. highly predictive, validly cued high-intensity targets. In these cases, presumably nicotine-induced recruitment of prefrontal regions was either insufficient to compensate for poor performance, or these hyperactivations reflected maladaptive responses that contributed to response slowing. Similar to points made in Section 6.2, of pro-cholinergic hypoactivations being associated with either performance improvement or deterioration (Jacobsen et al., 2004, 2006), the finding here of pro-cholinergic frontoparietal *hyperactivations* being associated with either positive or negative performance effects may also be attributed to task or subject differences, that entail different starting points on an inverted-U shaped profile of responses (see also Section 8).

In smokers, nicotinic stimulation appears to re-instate the normal prefrontal functional activation pattern whereby greater task demands (e.g. 3 > 2 > 1-back working memory) result in proportionate increases in activation (Loughead et al., 2010). However, this may occur either via drug-induced increases during the hardest condition (Loughead et al., 2009, 2010), or because of selective decreases during the easiest condition (Xu et al., 2005); in the latter case, the interpretation possibly being one of ‘enhanced efficiency’ (in line with Section 6.2). In non-smokers, nicotine may push subjects away from this normal recruitment pattern (Kumari et al., 2003), possibly because of excessive processing during easy conditions, and inappropriately diminished processing (or possibly exhaustion) during hard conditions (here again, reflecting an inverted-U shaped pattern of response).

Finally, the fact that cholinergic stimulants induce frontoparietal hyperactivations during a highly circumscribed set of task parameters, e.g. with spatial orienting rather than spatial working memory (Bentley et al., 2004); or intentional rather than attentional cues (Rose et al., 2010) argues against explanations in terms of the cholinergic system's proposed role in general arousal (see Section 1). This assertion is supported by data showing that frontoparietal hyperactivations secondary to cholinesterase inhibitors do not correlate with arousal or alertness (Bentley et al., 2004; Chuah and Chee, 2008), in contrast to activations induced by nicotine within the midbrain (Kumari et al., 2003).

## 7 Functional neuroimaging: memory modulations

### 7.1 Medial temporal regions

**Fig. 3:**
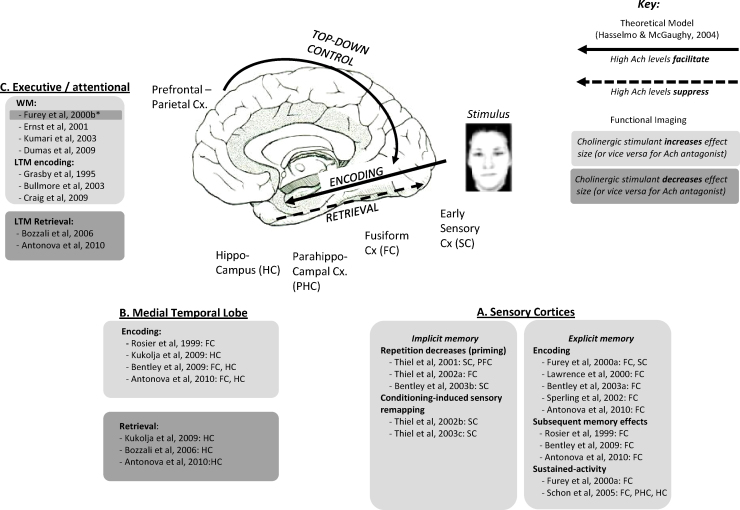
Overview of memory-related processes modulated by cholinergic drugs as revealed by cholinergic-functional imaging studies, and relationship with theoretical models in which high ACh levels facilitate encoding while suppressing retrieval (Hasselmo and McGaughy, 2004) as well as potentiate top-down control of sensory processing (Sarter et al., 2006). (A) Sensory regions, especially fusiform cortex, show enhanced activations with pro-cholinergic drugs (and vice versa with anti-cholinergics) during attention-demanding periods, including during encoding phases of working memory tasks, which correlates with subsequent memory. Sensory regions also demonstrate cholinergic sensitivity in several memory-related processes elicitable by functional imaging – sustained-activity, repetition decreases, and conditioning-induced sensory remapping. (B) Medial temporal regions show enhanced activation with pro-cholinergic therapies during encoding, but suppression during retrieval (or vice versa for anti-cholinergic therapies) – this profile corresponding to the discussed computational model of memory function. (C) Prefrontal regions show a similar pattern of responses as medial temporal regions: anti-cholinergic therapies decreasing activations during encoding or working memory paradigms, but increasing or not modulating activations during retrieval (and vice versa for pro-cholinergic therapies except in the case of one working memory paradigm* that was interpreted as increased efficiency – see Fig. 2B).

Given influences of cholinergic drugs, neuropathology and genes on memory performance (Kopelman, 1986; Anagnostaras et al., 2003), and the anatomical facts of an abundance of cholinergic terminals and receptors within rhinal–perirhinal cortex (Mesulam et al., 1986), it is reassuring that numerous neuroimaging studies demonstrate direct associations between cholinergic modulation of medial temporal structures and memory encoding (Fig. 3B and Table 5). Hence scopolamine reduces activation of hippocampal and parahippocampal cortices during encoding and maintenance phases of spatial (Antonova et al., 2010) and item (Sperling et al., 2002; Schon et al., 2005; Bozzali et al., 2006; Craig et al., 2010) paradigms, while often decreasing subsequent memory success. Conversely, cholinesterase inhibitors increase hippocampal responses to stimuli subsequently remembered compared to forgotten stimuli (Kukolja et al., 2009), and enhance associations between sensory cortex and hippocampal activations on trials subsequently remembered (Bentley et al., 2009), suggesting facilitation of neuronal encoding mechanisms that could account for these drugs’ pro-mnemonic actions. Indeed, in Alzheimer's disease or mild cognitive impairment, pro-cholinergic enhancements of memory-related hippocampal activity are even more apparent (Potkin et al., 2001; Goekoop et al., 2004; Grön et al., 2006; Teipel et al., 2006), and behavioural benefits are more manifest.

Attempts have been made to interpret cholinergic neuromodulations within medial temporal regions seen with functional imaging. Effects of scopolamine on working memory maintenance-period BOLD activity (Schon et al., 2005) have been interpreted in terms of persistent-spiking multi-unit activity, observed in perirhinal and entorhinal cortex neurons during, and after, encoding, e.g. in rats performing a delayed nonmatch-to-sample task (Young et al., 1997). Cortical slice studies have shown that this firing pattern is stimulus-specific and cholinergic-dependent (Egorov et al., 2002; Fransen et al., 2002). Furthermore, cholinergic-dependent, delay-period BOLD activity predicts not only working memory success, but also subsequent confident memory on a later surprise recognition test (Schon et al., 2005). This is consistent with models invoking persistent-spiking activity within hippocampus as being instrumental to encoding of long-term, recollection-based memory (Hasselmo and Wyble, 1997; Koene et al., 2003).

A further neurophysiological interpretation addresses the finding that responses in hippocampus to cholinergic challenge depend upon memory subcomponents. Thus, as well as *increasing* hippocampal responses to stimuli at *encoding*, physostigmine *decreases* activity in amygdala at *retrieval* (Kukolja et al., 2009), and, moreover, tends to worsen memory accuracy relative to placebo. Donepezil also selectively enhances hippocampal activity during stimulus presentation, while decreasing it at rest (Teipel et al., 2006). Scopolamine, on the other hand, decreases hippocampal activity at encoding (Craig et al., 2010), but increases amygdalar activity at retrieval (Antonova et al., 2010). It may also be relevant here that physostigmine increases hippocampal activations to successfully encoded locations, but decreases them to unsuccessfully encoded locations (Kukolja et al., 2009). Similarly, the direction that scopolamine influences hippocampal activations associated with subsequent memory depends upon whether the items are presented for a second time (Schon et al., 2005), with a decrease following single presentation (during which hippocampal activity is likely to be critical to recall), but increases when items are presented on a second time (so that on some trials, hippocampal activity during the initial presentation – that was measured – may in fact reflect encoding failure).

This profile of neuroimaging data mirrors behavioural findings: namely, scopolamine impairs memory when administered prior to, but not after, encoding (Ghoneim and Mewaldt, 1975; Atri et al., 2004; Rasch et al., 2006); whereas cholinesterase inhibition enhances encoding, but impairs retrieval (Rogers and Kesner, 2003; Gais and Born, 2004). Such state-dependent, bidirectional cholinergic influences have been explained in terms of differential ACh actions on cortical input type (Hasselmo and McGaughy, 2004) – with elevated ACh levels increasing feedforward activity – that encourages self-association of activated inputs, and therefore encoding, while decreasing feedback, retrieval-associated activity in medial temporal cortices. On this account, physostigmine would be expected to enhance novel stimulus-driven responses at encoding, but suppress responses to retrieval prompts of the same stimuli – as found in human hippocampus and amygdala (Kukolja et al., 2009). The fact that physostigmine-mediated hippocampal enhancements are only found to subsequently remembered stimuli (Kukolja et al., 2009; Bentley et al., 2009) suggest that these modulations are instrumental to any positive memory effect found with this drug class. This interpretation also fits with scopolamine generally decreasing hippocampal and amygdalar activations during encoding (e.g. Sperling et al., 2002; Craig et al., 2010; Dumas et al., 2010), often while impairing memory; but causing relative increases in these regions during retrieval (Antonova et al., 2010).

In the earlier discussion of attention-related cholinergic modulations (Section 6.3), we noted that pro-cholinergic drugs may *suppress* task-related activity, or enhance deactivations, in a ‘resting-state network’ that includes medial temporal regions (e.g. Furey et al., 2000a; Lawrence et al., 2002; Hahn et al., 2007). In these paradigms though, memory was not an explicit part of the task, and/or stimuli were symbolic, rather than rich in detail (e.g. scenes, faces) – both of which might be expected to engage perirhinal processing less than where pro-cholinergic drugs did increase activations in these areas (e.g. Schon et al., 2005; Kukolja et al., 2009). Hence the pattern of cholinergic modulation in medial temporal regions depends upon task (e.g. whether or not memory is an explicit aim), phase (e.g. encoding or retrieval) and the specific contrasts performed (e.g. whether as a function of subsequent memory, or task type). Hippocampal responses to cholinergic challenges may also interact with subject factors, e.g. age (Dumas et al., 2008, 2010) or estrogen levels (Craig et al., 2010).

Part of the reason for the high variability in cholinergic neuromodulations seen in functional imaging studies of the hippocampus may relate to the complex relationship between septohippocampal cholinergic system, hippocampus and memory. For example, systemic scopolamine can both increase hippocampal acetylcholine levels, while impairing memory function, possibly due to differential actions on pre- and postsynaptic muscarinic receptors, respectively (Mishima et al., 2000). Furthermore, while septohippocampal cholinergic levels correlate with memory performance, acetylcholine may not be necessary for hippocampal-dependent memory, with alternative circuits, or behavioural strategies, available if cholinergic inputs are selectively lesioned (Parent and Baxter, 2004). This may account for why cholinergic blockade impairs hippocampal activation during spatial memory encoding, while not affecting memory accuracy, and at the same time, increases activity within a frontal–neostriatal network (Antonova et al., 2010).

### 7.2 Sensory regions

Computational models of memory suggest that cholinergic facilitation of input-driven associativity within sensory cortices complement similar modulations within hippocampal–perirhinal cortices in supporting encoding and retrieval (Hasselmo and McGaughy, 2004). Functional neuroimaging studies support this by demonstrating that scopolamine suppresses hippocampal and fusiform cortex conjointly, specifically during visual memory-delay periods (Sperling et al., 2002; Bullmore et al., 2003; Schon et al., 2005); and impairs long-term fusiform cortex plasticity (Rosier et al., 1999); in both cases matched by impaired subsequent recognition (Fig. 3A). Conversely, physostigmine increases extrastriate visual activations during visual working memory delay-periods (Furey et al., 2000a), with greater modulation for longer delays (Furey et al., 2008a; Ricciardi et al., 2009), suggesting a cholinergic interaction with a memory, rather than merely sensory, process. A similar conclusion was reached by a study showing that physostigmine-induced increases in fusiform cortex activations during encoding correlate with subsequent memory success (Bentley et al., 2009). Presumably, recognised influences of ACh on neural processes such as feedforward associativity, long-term potentiation, and persistent-spiking, found within sensory as well as perirhinal–entorhinal cortices, may underlie many of these effects (Gu, 2003; Hasselmo and Stern, 2006).

Accounts of cholinergic influences on memory processes within sensory cortices need to dovetail with models of cholinergic impacts on attentional processing in similar regions (Sarter et al., 2005). In this regard, modelling has suggested that cholinergic influences on sensory cortex circuits – viz. enhancing feedforward relative to feedback connectivity (Gil et al., 1997) – serve both to enhance signal detection (and therefore certain aspects of attentional performance) and formation of novel input associations, likely to be critical for memory encoding (Hasselmo and McGaughy, 2004). One prediction then is that cholinergic modulations of memory will be greater during high- relative to low-attention conditions. Both psychopharmacological (Warburton et al., 2001; FitzGerald et al., 2008) and neuroimaging (Bentley et al., 2009) studies, employing depth-of-processing paradigms, support this with nicotine or cholinesterase inhibition boosting memory and fusiform cortex activations selectively for deeply, relative to superficially, encoded items. In other words, pro-cholinergic enhancements of sensory cortex activations that occur selectively during high-attention conditions (see Section 5.1), may facilitate subsequent recall of encoded stimuli. Additionally, cholinergic stimulation may enhance connectivity between sensory, hippocampal–amygdala and frontoparietal cortices (Bentley et al., 2009; Kobiella et al., 2011).

Cholinergic drugs also interact with two well-recognised functional imaging signatures of *implicit* memory within sensory cortices – viz. conditioning-associated sensory remapping, and repetition priming – often with congruent effects on behaviour (Table 3C and Fig. 3A). The neural correlates of this disruption suggest cholinergic influences on more than one sub-process. For example, disruption of priming by scopolamine manifests itself through a diminution of repetition suppression, by virtue of visual extrastriate cortex activation being *increased* selectively to old items under scopolamine (Thiel et al., 2001, 2002a), in contrast to reduction of activation with repetition under placebo. Given effects on behaviour are also selective for old items, this suggests that scopolamine reduces memory *storage* (i.e. maintenance of a particular representation), or *reactivation*, within sensory cortices. However, the additional findings of *reduced* new-item activity in prefrontal cortex (Thiel et al., 2001), and an absence of drug effect on priming if given after the item-study phase (Thiel et al., 2002c), suggests that *encoding* too may be disrupted, as is more generally recognised (Hasselmo and McGaughy, 2004).

While repetition suppression recorded electrically among monkey inferior temporal cortex neurons has not been found to be cholinergic-dependent (Miller and Desimone, 1993), the discrepancy with pharmacological-neuroimaging results may reflect restricted neural sampling, or shorter lag times, in the electrophysiological study. By contrast, the neuroimaging finding that scopolamine disrupts remapping of sensory cortex in the context of an auditory fear-conditioning paradigm (Thiel et al., 2002a) is remarkable in its accurate mirroring of results following cholinergic manipulation of similar sensory-learning paradigms in rodents (Weinberger, 2007).

Influences of pro-cholinergic drugs on implicit memory-related activations in sensory cortices appear mixed. On the one hand, physostigmine increases repetition suppression in higher visual cortex, specifically to attended items, with concordant effects on priming (Bentley et al., 2003a,b). This effect was due to drug-induced decreases to repeated stimuli (rather than increases to novel stimuli), and as such mirrors effects of scopolamine on repeated visual stimuli in a similar occipital region (Thiel et al., 2001). On the other hand, physostigmine impairs conditioning-related sensory remapping (Thiel et al., 2002b). However, unlike scopolamine – that reduces differential sensory responses by suppressing responses to *relevant* conditioned stimuli (CS+) (Thiel et al., 2002a) – physostigmine heightens responses specifically to *irrelevant* non-conditioned stimuli (CS−). Once again, both of these cholinergic-neuroimaging results (Bentley et al., 2003a,b; Thiel et al., 2002b) indicate that cholinergic modulation of sensory-based, memory processes interact with attention (Sarter et al., 2005).

One reason why physostigmine increased neuroimaging repetition suppression effects but decreased conditioning-associated sensory remapping may be on account of anatomical factors (see Section 5.2); with once again, inferior occipital–temporal regions responding to cholinergic stimulation with a heightening of attentional effects (typically seen in paradigms using face stimuli), and vice versa for lateral occipital–temporal regions (e.g. Bentley et al., 2003a; Furey et al., 2008a,b). Alternatively, physostigmine might impair differential activations in sensory cortex specifically during a conditioning paradigm because of the drug's tendency to increase ACh levels tonically, rather than phasically – which might encourage pairing of both CS+ and CS− stimuli with the unconditioned (i.e. noxious) stimulus, in the presence of high ACh levels (Thiel et al., 2002a).

### 7.3 Prefrontal regions

Cholinergic modulations of prefrontal activity during working memory tasks have been discussed earlier in the context of attentional effects (Sections 6.2 and 6.4), where it was noted that both cholinergic blockade and stimulation may decrease activity, albeit with different performance accompaniments (Fig. 3C). Prefrontal modulations related to long-term memory have also been observed, although, in an analogous pattern to cholinergic neuromodulation of medial temporal regions (Kukolja et al., 2009), effects vary depending on task and phase. Thus scopolamine-induced suppression of prefrontal cortex is associated with *impaired* performance when given *prior* to encoding (Sperling et al., 2002; Craig et al., 2009, 2010); but with *improved* performance when given *afterwards* (Bozzali et al., 2006). Prefrontal modulations may reflect both direct actions, e.g. due to scopolamine disrupting semantic processing of encoded words (Craig et al., 2009); and/or indirect actions, e.g. secondary to cholinergic potentiation of sensory or perirhinal cortices (Furey et al., 2008a).

Scopolamine-induced reductions of memory-related frontoparietal (and sensory) cortex activity, as well as of performance, resemble those induced by benzodiazepines within the same experimental paradigm (Thiel et al., 2001; Rosier et al., 1999; Sperling et al., 2002), implying a non-specific sedation effect. Arguing against this though is an absence of correlation between drug-induced modulations of memory-associated activation and vigilance scores (Thiel et al., 2001; Sperling et al., 2002). Strong interdependencies between cholinergic and GABAergic neurotransmission in many brain regions, including the septohippocampal pathway (Parent and Baxter, 2004), may account for such overlap in neuromodulatory responses between benzodiazepines and anti-cholinergics. Furthermore, the profile of behavioural and neural responses in a priming paradigm (Thiel et al., 2001) suggested a tendency for a greater relative effect of scopolamine on item storage, as opposed to lorazepam where effects appear to be on initial item encoding.

## 8 Inverted-U shaped patterns of cholinergic neuromodulations

**Fig. 4:**
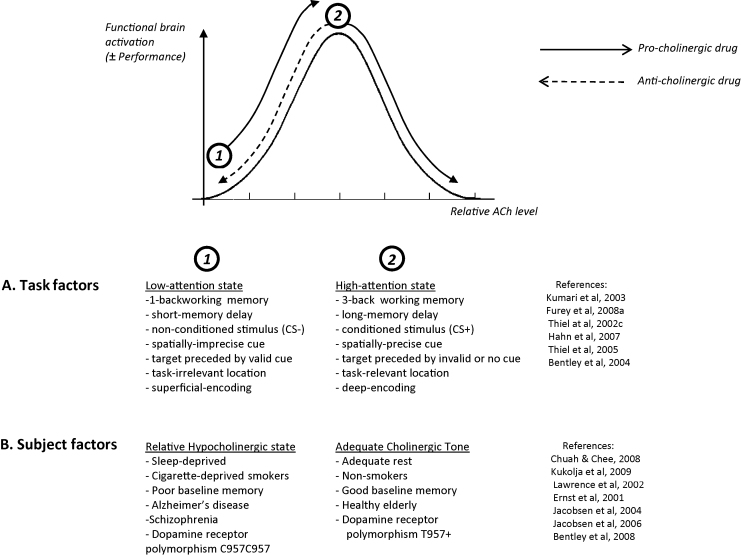
Modulations of functional imaging activations by cholinergic drugs often correspond to an inverted-U shaped pattern, depending upon both relative task demands (A) and subject-specific factors (B). In many cases, there is also a concordant effect on performance, e.g. in Alzheimer's disease where physostigmine increases task-related activations, reaction time and memory, or in healthy subjects where scopolamine decreases the same three parameters.

A finding across the literature as whole is that the pattern of cholinergic modulation often resembles an inverted U-shaped function that depends upon the level of regional activation *prior* to drug challenge (Fig. 4A). Thus, pro-cholinergic drugs enhance frontoparietal activity most readily under task conditions where such activity is relatively low under placebo; but decrease activity within the same regions, when activations are high to begin with (Kumari et al., 2003; Bentley et al., 2004; Furey et al., 2008a; Thiel et al., 2005); or conversely, diminish strong deactivations, while making weak deactivations more negative (Hahn et al., 2007, 2009). In many cases, low levels of activation (or weakly negative deactivations) under placebo, that strengthen with cholinergic stimulation, occur during low-attention conditions, e.g. during a 1-back working memory task (Kumari et al., 2003); superficial encoding (Bentley et al., 2008), or with poorly informative cues (Hahn et al., 2007) or validly cued targets (Thiel et al., 2005). Conversely, states with high levels of activation (or deactivation), that lessen with pro-cholinergic drugs, occur when attention is relatively higher, e.g. with a 3-back working memory task, deep encoding, or to highly informative cues, or invalidly cued, or uncued, targets (corresponding references as above). Similar inverted-U shaped cholinergic response profiles also occur in sensory (Hahn et al., 2007) and hippocampal (Schon et al., 2005; Kukolja et al., 2009) regions.

A related effect concerns examples where task-specific differential activation observed during unmedicated sessions decrease following treatment with *either* a cholinergic antagonist *or* cholinergic stimulant. As examples, working memory-associated prefrontal activity is suppressed by either physostigmine (Furey et al., 1997, 2000b), or scopolamine (Grasby et al., 1995; Dumas et al., 2008); while stimulus-evoked activations of primary visual cortex are suppressed either by donepezil (Silver et al., 2008) or scopolamine (Mentis et al., 2001). Furthermore, either scopolamine or physostigmine can decrease conditioning-associated sensory cortex remapping (Thiel et al., 2001, 2002c), and decrease lateral occipital–inferior temporal cortex activations during visual working memory paradigms (Bullmore et al., 2003; Freo et al., 2005).

One must consider how methodological issues might relate to putative inverted-U-shaped phenomena. For example, if a drug reduces all activations by 10%, then this may be more discernible for conditions with higher activations to begin with, due to the proportional effects being larger for those (and vice versa for drug-induced reductions of deactivations). Conversely, if the hemodynamic response to a particular condition is close to ceiling in some regions (due either to metabolic-vascular or neural limitations), then drug-induced increases in neural activity may only be manifest in other regions, or other conditions, where the hemodynamic response starts off low. This might explain, for example, why nicotinic effects in frontoparietal cortex during a working memory task are most apparent during a relatively easy 1-back, rather than more difficult, 3-back working memory conditions (Kumari et al., 2003; Xu et al., 2005); or why the enhancement of sensory cortex activity by physostigmine is more pronounced in task-irrelevant than task-relevant conditions (Bentley et al., 2004, 2008; Section 5.3). Furthermore, one must always be wary of ‘regression to the mean’ artefacts – arising from the fact that floor activations can only get higher, and ceiling activations only get lower. But such methodological considerations alone do not appear able to explain the full inverted U-shaped profile that is typically observed. For instance, while proportional changes can explain why a more activated region apparently shows stronger reduction in activation under a drug, this cannot explain ‘cross-over’ drug x condition interactions, for example, where the same region shows either an increase, or decrease, in response to a drug, depending on the starting activation level (e.g. Hahn et al., 2009). Moreover, concerns about regression to the mean are less likely in fully counterbalanced crossover designs, and/or by independent selection of regions of interest for analysis – techniques used in many of the quoted studies.

Might there be good neurobiological reasons that account for many of the inverted U-like profiles of response observed? According to the ‘attentional effort’ hypothesis (Sarter et al., 2006), endogenous neocortical cholinergic stimulation may be instrumental in enhancing anterior and posterior cortical activations in response to performance challenges. Consequently, exogenous pro-cholinergic drugs may mimic this modulatory effect, which can only be appreciated when activations are low to begin with – i.e. during undemanding conditions. Conversely, suppression of frontoparietal activity during high-attention conditions, and decreases in task-driven sensory cortex selectivity, with pro-cholinergic therapies may correspond to decreases in top-down or feedback processing, as commented upon earlier (Sections 2.1 and 2.2), in accord with existing models of cholinergic function (Hasselmo and McGaughy, 2004; Yu and Dayan, 2005).

A further type of inverted-U response, seen when comparing subject types (Fig. 4B), may also have a physiological basis. According to this profile, pro-cholinergic drugs normalize task-evoked activation levels in states – such as sleep-deprivation (Chuah and Chee, 2008), aging (Ricciardi et al., 2009) or disease (Blin et al., 1997; Jacobsen et al., 2004; Goekoop et al., 2006; Bentley et al., 2008), or with certain genetic polymorphisms (Jacobsen et al., 2006) – where such activations start off abnormally low or high. By contrast, many of these studies also show either no modulation, or a *reverse* pattern of modulation, in the same regions under the same paradigm, when healthy controls are tested with the same drugs. These contrasting neuromodulatory signatures for patients and controls are echoed by equivalent behavioural dissociations, with performance enhancements by pro-cholinergic drugs selectively in subjects with abnormal physiological states to begin with, but deteriorations instead for controls (Jacobsen et al., 2004; Bentley et al., 2008). This fits with data demonstrating that performance benefits of pro-cholinergic drugs are inversely correlated with baseline performance (Ernst et al., 2001b; Kukolja et al., 2009; Newhouse et al., 2004; Thiel et al., 2005; Beglinger et al., 2005). One explanation is that where drug-induced increases in activity and/or performance are observed, this reflects states in which there is a relative reduction in tonic ACh release prior to treatment, e.g. due to genetic variation, disease, sleep-deprivation or undemanding task conditions.

Responses to nicotine may also diverge depending upon subjects’ smoking status (e.g. Ernst et al., 2001a; Azizian et al., 2010; Rose et al., 2010). One of the reasons for this possibly relates to the fact that some smokers suffer adverse emotions and/or performance under placebo (i.e. abstinence from smoking), because of a dependency upon exogenous nicotine for normal mental well-being and cognitive performance, e.g. to compensate for chronic nicotinic receptor desensitization. Consequently, effects of nicotine, relative to placebo, in abstinent smokers may parallel the situation of cholinergic-deficient subjects, with pro-cholinergic therapies tending to normalize their usual level of cholinergic stimulation, and so ameliorate both aberrant neural responses (whether excessively high or low), and impaired performance. By contrast, nicotine given to non-smokers pushes subjects towards a hyper-cholinergic state relative to what they are accustomed to. This might explain why cholinergic stimulants increase fronto-parietal activity in smokers (e.g. Hong et al., 2009; Loughead et al., 2010), but has the opposite effect during similar tasks in non-smokers (Kumari et al., 2003; Furey et al., 2008a,b). Conversely, in non-smokers, nicotine increases prefrontal activity during an easy (1-back) condition (Kumari et al., 2003), whereas in smokers, it is the withdrawal of nicotine that heightens activity during this condition (Xu et al., 2005, 2006), possibly reflecting increased effort. Furthermore, the general pattern noted earlier of nicotine enhancing resting-state network deactivations, while increasing attention (Ernst et al., 2001a; Hahn et al., 2007), is sometimes reversed in smokers (Sweet et al., 2010), possibly because heightened attention is required during drug withdrawal and craving. Indeed, concentration difficulty following nicotine withdrawal is associated with withdrawal-associated changes in reciprocal-coupling between resting-state and executive control networks (Cole et al., 2010).

Inverted-U shaped functions are also seen with dopamine (Williams and Castner, 2006), and norepinephrine (Introini-Collison and McGaugh, 1986). For instance, amphetamine increases performance and prefrontal activation in subjects with low baseline measures of each, but decreases both in subjects who begin with high values for each (Mattay et al., 2000). Furthermore, differences in performance accounted for by genetic polymorphisms related to dopaminergic neurotransmission can produce an inverted-U pattern of response to nicotine (Jacobsen et al., 2006), providing evidence for cholinergic–dopaminergic interactions (Dewey et al., 1993). Thus, a common property of neuromodulators is that their process-optimising capabilities exist within a narrow concentration range. Two practical implications are that ‘performance-enhancing’ drugs may be less likely to benefit high-performers; and that the effects of such drugs may be predictable from individuals’ baseline behaviour or brain activity (Giessing et al., 2007).

## 9 Conclusion

Physiological consequences of ingesting cholinergic-active substances have been observed since the Ancient Greek era (Holzman, 1998), and have been instrumental both for understanding the natural cholinergic system, and for developing new pharmacological applications. Sophisticated experimental tools are now available that enable precise manipulation and measurement of cholinergic function, including cortical-slice recordings, the cholinergic-specific immunotoxin saporin, and choline microelectrodes (Hasselmo and Sarter, 2011).

The purpose of this review has been to evaluate the contribution made by human pharmacological whole-brain functional neuroimaging in relation to cholinergic physiology. While functional imaging is limited by its spatiotemporal imprecision (relative to more invasive methods) and its indirect relationship with neural activity, it has the notable advantages of being applicable noninvasively in humans, and of providing whole-brain coverage. Moreover multiple paradigms now exist where cholinergic pharmacological-neuroimaging findings closely mirror those from invasive studies. Notable examples discussed here are: disruption of auditory cortex remapping (Thiel et al., 2002a), repetition suppression (Thiel et al., 2001), and perirhinal memory-delay activity by scopolamine (Schon et al., 2005); as well as cholinergic stimulation causing a restriction of stimulus-induced propagation in visual cortex (Silver et al., 2008), and bidirectional responses in hippocampus and amygdala dependent upon the phase of memory processing (Kukolja et al., 2009). Importantly, the directions of such neuroimaging responses to cholinergic challenges, in the appropriate behavioural contexts, accord with analogous cholinergic manipulations at the electrophysiological level, but are now “scaled up” to a neural population level, and temporally blurred according to the hemodynamic or metabolic response functions.

Added value from functional imaging arises from its ability to test hypotheses using approaches more accessible than other techniques (e.g. by virtue of its whole-brain sampling; sensitivity to population activity; and use of more naturalistic paradigms than with non-human designs). For example, while interactions of ACh with attention have been described in isolated sensory neurons in vivo (Herrero et al., 2008), or for the effects of ACh on columnar excitability recorded in cortical slices (Kimura et al., 1999), functional imaging can complement these by assessing population activity in vivo, in parietal and sensory regions simultaneously, while orthogonally manipulating sensory and attentional variables. As summarised, imaging results also provide novel insights, while remaining consistent with extant models. Some of the most important examples here include: dependency on top-down influences for cholinergic modulation of sensory processing (e.g. Bentley et al., 2004); impairment of top-down, selective-attention effects in sensory cortices by either too much or too little cholinergic transmission (e.g. Bentley et al., 2008); the interactions of the above effects with uncertainty (e.g. probability of valid cuing: Giessing et al., 2006); cholinergic recruitment or downregulation of frontoparietal activations (e.g. Furey et al., 2008a,b), along with the possibility that acetylcholine interacts with the balance between task-related networks and the default or ‘resting-state’ network (Hahn et al., 2007). Many of these insights arise from the use of functional neuroimaging to study population-level, cortically distributed effects, that might be missed at the finer spatial grain targeted by single-unit or cortical-slice studies.

Having established that pharmacological-functional neuroimaging provides a meaningful tool for probing human neuromodulation, there follow several promising leads that lend themselves to future enquiry. First, given the likelihood that acetylcholine influence anatomically segregated but functionally interconnected regional processes, e.g. frontoparietal and sensory cortices (Sarter et al., 2001), it seems likely that many important neuromodulatory effects will be captured through study of changes in *inter-regional* effective connectivity (i.e. functional coupling), rather than through changes in the strength of *regional* activation per se. Analytic techniques for studying interplay between remote but interconnected regions (e.g. Friston et al., 2003) should ideally be used in conjunction with traditional contrast-based methods (e.g. Kobiella et al., 2011). Second, given concern over drug influences on the neurovascular relationship, and the possibility that this may vary anatomically and between patient groups, future pharmacological fMRI studies could usefully be supplemented by techniques such as arterial-spin labelling MRI (e.g. Franklin et al., 2011), that can assess regional blood flow, or techniques immune to vascular confounds such as magnetoencephalography (MEG). Third, radionuclide imaging techniques sensitive to an increasing array of cholinergic targets may shed further light on the exact mechanism of drug action and their localisation. Finally, by translating neurophysiological techniques from non-human to human subjects, we are in a stronger position to address questions regarding bridges between cholinergic models and clinical scenarios such as: how enhancement of task-irrelevant sensory activity by pro-cholinergic drugs relates to hypercholinergic models of anxiety and schizophrenia (Sarter et al., 2005); or how behavioural responses for particular drugs may be predictable by individual activation profiles during appropriate paradigms (Giessing et al., 2007).

## Conflict of interest

None declared.

## Figures and Tables

**Table 1:** Evidence for cholinergic interactions with neurovascular variables relevant to functional imaging.

**Anatomical**	
Cholinergic terminals apposed to cerebral cortex capillaries; arterioles and perivascular glia	Parnavelas et al. (1985), Arnerić et al. (1988), Chédotal et al. (1994)
**Cerebral blood flow (CBF)**	
ACh causes vasodilatation	Furchgott and Zawadzki (1980)
ACh increases CBF	Scremin et al. (1973), Matsuda et al. (1976)
Nicotine or cholinesterase inhibition causes cerebral vasodilation and increased CBF	Linville et al. (1993), Uchida et al. (1997), Aoyagi et al. (1975), Nakahata et al. (2008)
CBF increase can occur through stimulation of cortical cholinergic interneurons or nucleus basalis	Scremin et al. (1991), Fukuyama et al. (1996), Biesold et al. (1989)
**CBF variability**	
ACh effects on CBF are region-dependent	Sato et al. (2004), Lacombe et al. (1989)
ACh effects on CBF are age-dependent	Uchida and Hotta (2009)
**Neurovascular coupling**	
Scopolamine impairs functional hyperemia but not cerebral glucose metabolism	Ogawa et al. (1994)
Cholinergic lesions impair cerebral glucose metabolism without affecting rCBF response	Ouchi et al. (1996), Ogawa et al. (1996)
Physostigmine increases CBF without altering cerebral oxygen consumption	Scremin et al. (1982), Tsukada et al. (1997, 2000)
Physostigmine increases global CBF while decreasing regional glucose metabolism	Blin et al. (1997)^a^
Nucleus basalis stimulation increases cortical ACh release and CBF but not cerebral metabolism or EEG changes	Kimura et al. (1990), Hallstrom et al. (1990), Lacombe et al. (1989), Vaucher et al. (1997)
**Baseline activity**	
Cholinergic drugs affect baseline pattern of activity	McNamara et al. (1990), Gustafson et al. (1987)^a^, Gitelman and Prohovnik (1992)^a^, Blin et al. (1994)^a^, Stein et al. (1998)^a^

a Human studies.

**Table 2:** Methodological strategies to check for neurovascular confounding in pharmacological-functional imaging studies.

	Example references
**Global physiological–psychological indices**	
Systemic physiological measurements – pulse, blood pressure: compare between sessions; or include as regressors of no interest	Kukolja et al. (2009)
Subjective scores (e.g. Bond and Lader, 1974) – as for physiological parameters	Thiel et al. (2001)
Co-administration of drug that counteracts extracerebral cardiovascular side-effects, e.g. glycopyrrolate with centrally acting cholinesterase inhibitor	Furey et al. (2000a)
**Mean rCBF/BOLD values**	
Grand mean rCBF or BOLD values – over whole brain per *session*; compare between sessions and/or correct (scaling)	Grasby et al. (1995); most fMRI studies
Global mean BOLD values – over whole brain per *scan*; include as regressor of no interest or correct (scaling)	Thiel et al. (2001)
High-pass filtering – removes gradual changes in response, e.g. due to declining drug levels	Most fMRI studies
Voxel-level session effect; compare across conditions	Bentley et al. (2004)
**Drug** **×** **condition interactions**	
Dissociations: identify interactions with similar activation levels between conditions under placebo (e.g. placebo: A – low; B – low; drug: A – low; B – high)	Bentley et al. (2004)
Cross-over: identify interactions in which drug causes an opposite pattern of responses across conditions (i.e. placebo: A – high; B – low; drug: A – low; B – high)	Hahn et al. (2007), Kukolja et al. (2009)
Cross-over re-mapping: e.g. where drug modulates differential responses to two arbitrary stimuli whose cognitive significance reverses in half of subjects	Thiel et al. (2002a)
Region-specific interactions, e.g. task-specific drug interaction in parietal but not occipital cortex despite similar activation levels under placebo	Thiel et al. (2001), Sperling et al. (2002)
**Behavioral correlations**	
Correlation of drug-induced activation change and behavioural measure of interest suggests drug effect on neural activity, especially if scan and behavioural measure separated in time	Furey et al. (2000b), Bentley et al. (2009)
**Alternative analytic methods**	
Modelling multiple basis functions for BOLD response	Rombouts et al. (2005)
Measurement of BOLD phase relationship only with respect to alternating visual stimulus	Silver et al. (2008)
Fractal complexity (Hurst exponent, H) and inter-regional correlations of resting-state fMRI time-series	Wink et al. (2006), Suckling et al. (2008)
Inter-regional correlations of event-related fMRI (functional connectivity)	Jacobsen et al. (2004), Kobiella et al. (2011)
**Measurement of BOLD and rCBF**	
Use of arterial-spin labelling in addition to T2* MRI sequences	Hahn et al. (2009)

**Table 3:** Cholinergic functional imaging studies – sensory cortices.

	Scanning task	Drug	Effect of drug on functional activations	Effect of drug on performance
**A. No task/irrelevant task/task-independent**				
Cohen et al. (1994) PET-FDG	Auditory discrimination	Scopolamine	↓ primary visual, parieto-occipital cx (i.e. irrelevant sensory cx); N.B. no control task	Poorer target discrimination. Performance inversely correlated with parieto-occipital cx activity
Grasby et al. (1995) PET-rCBF	Auditory word: 5- and 15-spans	Scopolamine	↑ bilateral lateral occipital cx (i.e. irrelevant sensory cx), in sub- and suprascan tasks	Memory impairment on supraspan task only
Bahro et al. (1999) PET-rCBF	Auditory – eyeblink conditioning	Scopolamine	↑ lateral occipital–temporal cx (i.e. irrelevant sensory cx); N.B. no direct comparison with placebo group	Not measured
Thiel et al. (2001)	Word-stem completion	Scopolamine	No effect in primary visual cortex across task conditions	No effect on performance independent of repetition
Sperling et al. (2002)	Face–name pairs	Scopolamine	No effect in primary visual cortex across task conditions	Memory impaired
Jacobsen et al. (2002)^a^	Chequerboard	Nicotine	No effect in sensory cortices	Not measured
Hahn et al. (2007)^a^	Chequerboard	Nicotine	No effect in sensory cortices	Not measured
Hahn et al. (2009)^a^	Chequerboard	Nicotine	No effect in sensory cortices	Not measured
Mentis et al. (2001) PET-rCBF	Alternating eye light flash	Physostigmine ± scopolamine	Physostigmine: ↓ middle occipital Physo. + scopolamine: ↑ middle occipital No effect of physo. in primary visual cx Physo + scopolamine: ↓ primary visual cx	Not measured
Furey et al. (2000a)	Face WM	Physostigmine	No effect on control stimuli in extrastriate cx	Not measured
Silver et al. (2008)	Chequerboard	Donepezil	↓ primary visual cortex extent and magnitude	Not measured
Bentley et al. (2004)	Chequerboard	Physostigmine	↓ primary visual cortex across task conditions	Faster RT over all tasks independent of task
Bentley et al. (2008) (Elderly)	Face versus houses	Physostigmine	↓ fusiform, parahippocampal cx, across task conditions	No task-independent effect
**B. Demanding perceptual task**				
Thienel et al. (2009a)	Attention network task	Mecamylamine	↓ superior occipital cx; ↑ anterior fusiform cx – orienting; ↓ calcarine cx – conflict	Slowing across all trial types; no interactions
Thienel et al. (2009b)	ANT	Scopolamine	↑ middle occipital cx – alerting; ↓ lingual gyrus, inf temporal cx – conflict	Slowing of responses
Ghatan et al. (1998) PET-rCBF^a^	Visual maze	Nicotine	↑ occipital–temporal–parietal cx more during difficult than control task	No effect
Thiel et al. (2005)	Alerting/spatial cues	Nicotine	↓ lateral occip.–temp., medial occip. – alerting; ↓ post. occip, post. fusiform cx, but ↑ anterior occip., anterior fusiform cx – orienting	Speeding of invalidly cued trials; alerting numerically but insignificantly speeded
Hahn et al. (2007)^a^	Spatial cues	Nicotine (smokers)	↑ cuneus (valid precise-cues), ↓ cuneus (valid imprecise-cues); ↑ lingual gyrus (invalid low-intensity targets); ↓ lingual gyrus (invalid high-intensity targets)	Speeding in precise-cueing trials
Thiel and Fink (2008)	Spatial cues	Nicotine	No effect in occipital cx	Less slowing in invalidly cued trials
Vossel et al. (2008)	Spatial cues	Nicotine	↓ anterior lingual gyrus to invalid versus valid cues in high versus low-predictability blocks	Reduced invalidity effect
Hahn et al. (2009)	Visual angle; colour; signal-detection	Nicotine (smokers)	↓ occipital–temporal cx across all tasks (i.e. high- and low-attention)	Speeding in selective-attention and signal-detection tasks, but not divided attention
Loughead et al. (2011)^a^	Emotion detection	Varenicline for 13 days	↓ middle occipital cx	Speeded responses
Bentley et al. (2003a)	Spatial cues	Physostigmine	↑ fusiform cx; ↓ lateral occipital cx	Trend for speeded responses
Bentley et al. (2004)	Spatial cues	Physostigmine	↑ superior, lateral occipital cx; ↓cue-driven, differential retinotopic activity	Speeded responses. ↓ cue-driven occipital selectivity correlates with ↓ invalidity effect
Bentley et al. (2008) (Elderly)	Visual depth of processing	Physostigmine	↓ task-differential activity in posterior STS, lateral occipital cx, due to ↑ activity in low-attention task	No effect
**C. Memory (encoding)**				
Rosier et al. (1999) PET-rCBF	Shape recognition	Scopolamine (at encoding); scan 3 days later	↓ bilateral fusiform cx, esp. L (both tasks), and middle occipital cx (during sensory-challenge rather than standard conditions)	Impaired recognition accuracy. Fusiform activity correlates with memory accuracy
Sperling et al., 2002	Face–name pairs	Scopolamine	↓ fusiform cx	Activity correlates with subsequent memory
Bullmore et al. (2003)	Object–location	Scopolamine	↓ lateral occipital; inferior temporal; cuneus during task independent of memory load	No effect
Schon et al., 2005	Delayed match-to-sample	Scopolamine	↓ bilat. mid-fusiform, parahippocampus (delay-period of WM); ↓ R fusiform (delay-period of subsequently remembered trials)	Impairs performance on control task, WM task and subsequent memory
Dumas et al. (2010)	Word recognition	Scopolamine/mecamylamine	↓ cuneus, lat. occip. (scopolamine), middle occip. (mecamylamine): new versus old words	Worse recognition with scopolamine (trend)
Antonova et al. (2010)	Allocentric spatial WM	Scopolamine	↓ fusiform cx at encoding	No effect
Lawrence et al. (2002)^a^	Visual number WM (RVIP)	Nicotine	↑ middle occipital, fusiform cx in RVIP and visuomotor control task	Improved accuracy on RVIP task (dependent on treatment order)
Hong et al. (2009)^a^	RVIP	Nicotine	↑ cuneus, fusiform, parahippocampal cx	Improved accuracy on RVIP task
Jacobsen et al. (2004)^a^	Auditory n-back; dichotic versus binaural	Nicotine	↑ posterior sup. temporal cx during 2-back, not 1-back; ↓ medial occipital (i.e. irrelevant sensory cx) during dichotic presentation	Accuracy worsened in hardest condition (2-back, dichotic)
Jacobsen et al. (2006)^a^	Auditory n-back	Nicotine	↓ sup. temporal cx during 2-back, dichotic; ↓ occip., fusiform (i.e. irrelevant sensory cx)	Accuracy worsened in hardest condition (2-back, dichotic)
Furey et al. (1997) PET-rCBF	Face WM	Physostigmine	↓ lateral temporo-occipital cx in WM versus control tasks	Speeded responses
Furey et al. (2000b) PET-rCBF	Face WM	Physostigmine	↑ medial occipital correlates with RT decreases	Speeded responses, and correlation with activation increases
Furey et al. (2000a)	Face WM	Physostigmine	↑ amplitude in fusiform, occipital, parietal cx (encoding phase); ↑ activation volume in occipital, inf temporal cx (encoding and delay)	Trend to speeded responses
Bentley et al. (2004)	Spatial WM	Physostigmine	↑ middle, superior occipital cx during encoding phase only	Speeding over all tasks. No effect on accuracy
Freo et al. (2005) PET-rCBF	Face WM	Physostigmine	↑ medial occipital (in elderly); ↓ es lateral occipital, ventral temporal cx (esp in young)	Speeded responses
Furey et al. (2008a,b) PET-rCBF	Face WM	Physostigmine	↓ lateral occipital cx (1, 6, 16 s delays); ↑ medial occipital cx (6–16 s delays)	Speeded responses independent of delay
Chuah and Chee (2008) (sleep-deprived)	Visual color WM	Donepezil	↑ visual extrastriate cx in sleep-deprived, independent of item number	Improved performance; correlated with activation enhancements
Ricciardi et al. (2009) PET-rCBF (Young, elderly)	Face WM	Physostigmine	↓ lateral occip., ventral temporal (young); ↑ lateral occip. (elderly): for long delays; ↑ medial occipital cx (all): for long delays	Speeded responses independent of delay
Bentley et al. (2009) (Elderly)	Face recognition	Physostigmine	↑ fusiform cx correlating with memory of deep versus superficial encoded faces	Correlates with memory improvement
**D. Memory (conditioning)**				
Thiel et al. (2002a)	Auditory fear conditioning	Scopolamine	↓ auditory cx plasticity due to ↓ response to CS+ or ↑ response to CS−	Reduced speeding of responses to CS+ (paired) relative to CS− (unpaired tone)
Thiel et al. (2002b)	Auditory fear conditioning	Physostigmine	↓ auditory cx plasticity due to ↑ CS− response (unpaired tone)	No effect
**E. Memory (priming)**				
Thiel et al. (2001)	Word stem-cell completion	Scopolamine	↓ L lateral occipital repetition decrease due to ↑ response to repeated stimulus. No effect in primary visual cortex.	Reduced priming (accuracy) for previously presented words
Thiel et al. (2002c)	Faces – judging famousness	Scopolamine	↓ fusiform cx (all faces); ↓ fusiform repetition decrease (famous faces), due to higher signal with repeated face	Reduced priming (RT) for repeated famous faces; no effect if drug given after study phase
Bentley et al. (2003b)	Spatial attention using faces, houses	Physostigmine	↑ repetition decreases only to attended faces, due to larger ↓ during repeated face	↑ priming effect for attended versus unattended faces

N.B.: All studies use BOLD-fMRI except where indicated under study first author. *Abbreviations*: WM, working memory; ANT, attention network task; RVIP, rapid visual information processing task; cx, cortex; PFC, prefrontal cortex; RT, reaction time; sup., superior; post., posterior; occip., occipital; temp., temporal.

a Subjects included smokers.

**Table 4:** Cholinergic functional imaging studies – task-related activations in frontal, parietal, lateral temporal cortices, and subcortical regions.

	Scanning task	Drug	Effect of drug on functional activations	Effect of drug on performance
**A. Sensory – passive/task**				
Cohen et al. (1994) PET-FDG	Auditory discrimination	Scopolamine	↓ thalamus, R PFC, cingulate, inf parietal cx; ↑ L anterior prefrontal, superior parietal cx	Poorer discrimination of targets. Correlation between R PFC and score
Thienel et al. (2009a)	Attention network task	Mecamylamine	↑ OFC (alerting); ↓ sup. PFC (orientation); ↑ sup. PFC (no-orientation trials); ↓ precuneus, sup. parietal (conflict); ↑ L inf. parietal (conflict)	Slowing across all trial types; no interactions
Thienel et al. (2009b)	Attention network task	Scopolamine	↑ R middle temporal; ↑ L sup. PFC (alerting); ↓ L sup. PFC (orientation); ↓ ant. cing., OFC, R PFC, precuneus (conflict); ↑ L inf. parietal (conflict)	Slowing across all trial types; greater slowing for incongruent (conflict) trials; also reduced interaction of alerting with conflict
Ghatan et al. (1998) PET-rCBF^a^	Visual maze	Nicotine	↓ ant. cing., basal ganglia, thalamus, cbllm	No effect
Mentis et al. (2001) PET-rCBF	Alternating eye light flash	Physostigmine ± scopolamine	Physostigmine: ↓ inf. parietal; ↑ thalamus; scopolamine: no effect in these regions	Not measured
Thiel et al. (2005)	Alerting/spatial cues	Nicotine	↑ R angular gyrus, R PFC (alerting); ↓ L lateral occipito-temporal during alerting; ↓ L parietal, precuneus during invalid-cue	Speeding of invalidly cued trials, esp in subjects with large validity effect at baseline
Giessing et al. (2006)	Visual spatial cues	Nicotine	↓ R post. parietal (invalid cues, highly reliable); ↑ R post. parietal (valid cues; poorly reliable)	No effect
Thiel and Fink (2007)	Auditory/visual alerting	Nicotine	↓ R parieto-occipital, frontal, sup temporal, ant. cingulate (cued trials); ↑ R angular gyrus (cued visual trials); ↓ R angular gyrus (uncued trials)	Trend to speeding for cued visual trials and uncued auditory trials
Xu et al. (2007)^a^	Stroop color test	Nicotine (smoking)	↓ R precentral sulcus during incongruent condition (i.e. reverses abnormal hyperactivation not seen in non-smokers)	No effect
Kobiella et al. (2011)	Passive viewing of emotional stimuli	Nicotine	↑ anterior cingulate, OFC, striatum for unpleasant (versus pleasant) stimuli; ↑ es coupling between ant. cingulate and amygdala	No effect on subsequent memory
Thiel and Fink (2008)	Spatial cues	Nicotine	↓ R parietal, L inf. PFC, temporal (invalid trials)	Speeding of invalidly cued trials
Vossel et al. (2008)	Spatial cues	Nicotine	↓ R parietal, temporal, ant. cing. (invalid trials, 90%-reliable); ↑ R parietal (invalid trials, 60%-reliable)	Speeding of invalidly cued trials in 90%-valid block, but slight slowing in 60%-valid block
Hahn et al. (2007)^a^	Spatial cues	Nicotine	Enhances deactivations in ant. and post. cingulate, L angular gyrus, L PFC. ↑ R PFC; ↓ thalamus (valid targets); ↓ precuneus (invalid targets); ↓ R PFC, L parietal (invalid; high-intensity)	Speeding for precise-cue, high-intensity targets, and invalid trials. Improved accuracy with high-intensity targets. Correlation of RT ↓ and nicotine-induced BOLD deactivations
Hahn et al. (2009)^a^	Visual angle; colour sequence; signal-detection	Nicotine	↓ dorsal prefrontal during low-attention, but ↑ during high-attention; also main-effect ↓ (enhances deactivation) in ant. cing., medial PFC, parahippocampal cx	Speeding of high and low-attention tasks. Correlations of RT ↓ with thalamus, PFC deactivations in signal-detection task
Ettinger et al. (2009)^a^	Pro- and anti-saccades	Nicotine	↓ dorsal prefrontal during anti-saccades; ↓ posterior cingulate, precuneus, R superior temporal gyrus during pro-saccades	Speeding of anti-saccades
Azizian et al. (2010)^a^	Color-word Stroop task	Nicotine (smoking)	↓ anterior cingulate during incongruent trials; ↑ middle frontal	Speeding independent of congruency
Rose et al. (2010)^a^	Intention versus Attention Cues	Nicotine	↑ L parietal, R superior temporal gyrus, to intentional, but ↓ for attentional, cues	Improved accuracy for both cue types
Franklin et al. (2011) MRI-perfusion^a^	Passive viewing smoking cues	Varenicline for 3 weeks	↓ medial OFC, ventral striatum to smoking cues, but ↑ lateral OFC	Reduced withdrawal symptoms while viewing smoking cues
Loughead et al. (2011)^a^	Emotion detection	Varenicline for 13 days	↓ medial prefrontal, cingulate cx, thalamus; ↑ middle temporal gyrus	Speeding responses
Bentley et al. (2004)	Spatial cues	Physostigmine	↑ superior prefrontal cx; ↓ medial parietal cx	Speeding and improved accuracy
Bentley et al. (2008) (Elderly)	Visual depth of processing	Physostigmine	↓ R parietal cx	No effect
**B. Working memory**				
Grasby et al. (1995) PET-rCBF	Auditory word lists: 5- and 15-words	Scopolamine	↓ bilat. PFC, ant. cing. (supraspan task); ↓ premotor, R thalamus, precuneus; ↑ OFC in supra- and subspan tasks	Memory impairment on supraspan task only
Dumas et al. (2008) (Elderly)	Visual verbal n-back WM	Scopolamine/mecamylamine	↓ R prefrontal (either drug); ↓ precuneus (scopolamine)	No effect
Craig et al. (2010) (Menopause)	Delayed match-to-sample WM	Scopolamine (±GnRH)	↓ bilat. PFC, anterior cingulate, R parietal, especially in GnRH group	Poorer accuracy and slower, especially in GnRH group
Antonova et al. (2010)	Allocentric spatial WM	Scopolamine	↓ L lateral temporal cx; ↑ PFC, cingulate, parietal, striatum, thalamus (mostly at recall)	No effect
Ernst et al. (2001a) PET-rCBF^a^	Visual letter 2-back WM	Nicotine	↑ L lateral PFC; bilat. parietal cx; ↓ ant. cingulate (in ex-smokers); ↓ frontoparietal, ant. cingulate (smokers)	Improves accuracy in smokers; accuracy correlates positively with PFC, cingulate activity under nicotine
Lawrence et al. (2002)^a^	RVIP and target detection	Nicotine	↑ bilat. parietal, post. cingulate, caudate, thalamus (RVIP); enhances insula deactivations	Improved accuracy on RVIP task (dependent on treatment order)
Hong et al. (2009)^a^	RVIP	Nicotine	↑ bilat. prefrontal, cingulate, parietal cx; insula, thalamus; striatum; midbrain, cbllm.	Improved accuracy on RVIP task; correlated with ↑ BOLD activity
Kumari et al. (2003)	n-Back WM	Nicotine	↑ dorsofronto-parietal, ant. cingulate, esp at 1-back; ↓ R dorsal parietal for 3-back	Increased accuracy, and correlation with BOLD effects. RT ↓ in 3-back
Jacobsen et al. (2004)^a^	Auditory 1- or 2-back	Nicotine	↓ R frontal, pallidum and thalamus during dichotic (high-attention) or 2-back conditions	Impaired accuracy during dichotic, 2-back condition
Jacobsen et al. (2006)^a^	Auditory 1- or 2-back	Nicotine	↓ L prefrontal, posterior cingulate during dichotic 2-back condition	Impaired accuracy during dichotic, 2-back (more so in 957T carriers)
Xu et al. (2005)^a^	n-Back WM	Nicotine (smoking)	↓ L dorsolateral PFC during 1-back (but not 2-back or 3-back)	Slower and less accurate with abstinence (trends)
Xu et al. (2006)^a^	n-Back WM	Nicotine (smoking)	↓ L dorsolateral PFC during 1-back (during abstinence day) but ↑ PFC during 1-back (during ad libitum smoking day)	Improved accuracy (trend)
Sweet et al. (2010)^a^	2-Back verbal WM	Nicotine	↑ inferior, middle temporal; parietal (correlations with craving during placebo); ↓ deactivations in medial PFC, temporal poles	No effect
Sutherland et al. (2011)^a^	Attention-switching WM	Nicotine (smoking)	No effects with acute challenge (but higher PFC activity in smokers than non-smokers)	Speeded responses and more accurate in smokers
Loughead et al. (2010)^a^	Visual pattern n-back WM	Varenicline (in abstinent smokers)	↑ prefrontal cx during 3-back	Speeded responses with drug in highly dependent smokers across all conditions
Furey et al. (1997) PET-rCBF	Face WM	Physostigmine	↓ R prefrontal cx	Speeded responses and correlation with prefrontal reductions
Furey et al. (2000b) PET-rCBF	Face WM	Physostigmine	↓ R prefrontal cx ant. cingulate, L lateral temporal cx correlates with RT decreases	Speeded responses and correlations with activation decreases
Furey et al. (2000c) PET-rCBF	Face WM	Physostigmine	↓ R prefrontal cx	Speeded responses
Furey et al. (2000a)	Face WM	Physostigmine	↓ anterior dorsal prefrontal cx,; ↑ inferior PFC, to all phases of task	Speeded responses
Freo et al. (2005) PET-rCBF (Young, elderly)	Face WM	Physostigmine	↓ dorsal (young) and anterior, inferior (elderly) PFC; trend to ↑ in ant. cingulate cx; greater deactivations in insula, medial frontal	Speeded responses in both young and elderly
Furey et al. (2008a,b) PET-rCBF	Face WM	Physostigmine	↓ anterior, inferior prefrontal cx, esp. at longer WM delays; ↓ sup. PFC at all delays	Speeded responses independent of delay
Ricciardi et al. (2009) PET-rCBF	Face WM	Physostigmine	↓ anterior prefrontal cx	Speeded responses independent of delay
Chuah and Chee (2008) (sleep-deprived)	Visual color WM	Donepezil	↑ R intraparietal sulcus. L prefrontal in sleep-deprived	Improved performance; correlated with activation enhancements
Bentley et al. (2004)	Spatial WM	Physostigmine	↓ L inferior prefrontal cx	Speeding. No effect on accuracy.
**C. Short-term memory**				
Rosier et al. (1999) PET-rCBF	Shape recognition	Scopolamine: – at encoding; scan 3 days later	↑ posterior thalamus, bilateral parietal	Impaired recognition accuracy. No effect on stimulus discrimination or detection (at time drug given)
Thiel et al. (2001)	Word stem-cell completion	Scopolamine	↓ inferior and middle PFC repetition decrease due to ↓ response to new items	Reduced priming for previously presented words
Sperling et al. (2002)	Face–name pairs	Scopolamine	↓ inferior, dorsolateral, orbital PFC; deactivations in lateral parietal, precuneus, lateral temporal cx	Slowed responses to gender judgement. Impaired subsequent memory.
Bullmore et al. (2003)	Object–location learning	Scopolamine	↓ bilateral dorsolateral PFC, ant. cingulate, striatum for high memory loads; ↓ parietal for high and low memory loads	No effect
Bozzali et al. (2006)	Word retrieval	Scopolamine	↓ bilateral PFC in exclusion condition (i.e. source not familiarity memory) for New but not old items	No overall effect. Correlation of ↓ in left PFC activity with score on New items
Craig et al. (2009) (Menopause)	Subsequent memory for written words	Scopolamine (±GnRH)	↓ L inferior frontal cx subsequent memory effect in subgroup treated with GnRH	Impaired recognition
Dumas et al. (2010)	Word recognition memory	Scopolamine/mecamylamine	↓ parietal cx (either drug); ↑ frontal cx (either drugs; trends), for new versus old words	Worse recognition with scopolamine (trend)

*Abbreviations*: RT, reaction time; WM, working memory; ANT, attention network task; RVIP, rapid visual information processing task; cx, cortex; PFC, prefrontal cortex; OFC, orbitofrontal cortex; cing., cingulate; bilat., bilateral; ant., anterior; post., posterior; sup., superior; inf., inferior; cbllm, cerebellum; GnRH, gonadotrophin releasing hormone (decreases estrogen secretion and so mimics menopause).

a Subjects included smokers.

**Table 5:** Cholinergic functional imaging studies – medial temporal areas.

	Scanning task	Drug	Effect of drug on functional activations	Effect of drug on performance
**A. Memory**				
Sperling et al. (2002)	Face–name pairs	Scopolamine	↓es fusiform cx, anterior hippocampus	Correlates with memory impairment
Bullmore et al. (2003)	Object–locations	Scopolamine	↓ hippocampal, parahippocampal cx. For higher memory load	No effect
Schon et al. (2005)	Scenes: delayed match-to-sample WM; subsequent memory test	Scopolamine	↓ fusiform, parahippocampal (WM delay). ↓ fusiform, parahippocampal, hippocampus (WM delay, for subsequently remembered items presented once); ↑ hippocampus (subsequent memory effect for stimuli previously presented twice)	Impairs accuracy and speed on visual control task and WM task. Impairs subsequent confident memory
Bozzali et al. (2006)	Word retrieval	Scopolamine	↓ L perirhinal cx in exclusion condition (i.e. source not familiarity memory) for new but not old items	No overall effect. Correlation of ↓ in left perirhinal cx activity with score on new items
Dumas et al. (2008) (Elderly)	Visual verbal n-back WM	Scopolamine/mecamylamine	↑ R parahippocampal cx (mecamylamine)	No effect
Dumas et al. (2010) (Elderly)	Word recognition memory	Scopolamine/mecamylamine	↓ R uncus (scopolamine); L parahippocampal cx (mecamylamine, trend); ↑ R hippocampus (either drug, trend)	Worse recognition with scopolamine (trend)
Craig et al. (2010) (Menopause)	Delayed match-to-sample WM	Scopolamine	↓ L parahippocampal cx, during encoding, especially in GnRH group	Poorer accuracy and slower, especially in GnRH group
Antonova et al. (2010)	Allocentric spatial memory	Scopolamine	↓ hippocampal, parahippocampal cx (at encoding); reduces deactivations in amygdala (at encoding and recall)	No effect
Postma et al. (2006)	Tactile pre-pulse inhibition	Nicotine	↑ hippocampus in prepulse + pulse verus pulse only	Increases pre-pulse inhibition
Furey et al. (2000a)	Face WM	Physostigmine	↓ L hippocampus correlates with RT ↓	Speeding of responses
Bentley et al. (2009) (Elderly)	Depth of processing face memory	Physostigmine	No effect on subsequent-memory comparison; enhanced correlation between hippocampal successful encoding and fusiform cx	Increased depth of processing
Kukolja et al. (2009)	Item and spatial source memory	Physostigmine	↑ R hippocampal (successful encoding); ↓ R amygdala (encoding); ↓ R amygdala (successful retrieval)	↓ in source memory accuracy (trend); Baseline memory negatively correlated with physostigmine effect on memory
**B. Other tasks**				
Thienel et al. (2009a)	ANT	Mecamylamine	↑ L parahippocampal cx during orienting	Slowing of responses
Thienel et al. (2009b)	ANT	Scopolamine	↓ L hippocampus during alerting	Slowing of responses
Lawrence et al. (2002)^a^	RVIP	Nicotine	Enhances L parahippocampal, amygdala deactivations	Improved performance
Hong et al. (2009)^a^	RVIP	Nicotine	↑ parahippocampal cx	Improved accuracy
Vossel et al. (2008)	Spatial cues	Nicotine	↓ R hippocampus to invalid versus valid cues	Reduced invalidity effect
Hahn et al. (2009)^a^	Several attention tasks	Nicotine	Enhances L parahippocampal deactivations	Speeding of responses
Kobiella et al. (2011)	Viewing emotional stimuli	Nicotine	↑ amygdala, hippocampus for unpleasant (rather than pleasant) stimuli; ↑ coupling between ant. cingulate and amygdala	No effect on subsequent memory

*Abbreviations*: WM, working memory; ANT, attention network task; RVIP, rapid visual information processing task; cx, cortex; PFC, prefrontal cortex; GnRH, gonadotrophin releasing hormone (decreases estrogen secretion).

a Subjects included smokers.
